# Construction of a prognostic model with CAFs for predicting the prognosis and immunotherapeutic response of lung squamous cell carcinoma

**DOI:** 10.1111/jcmm.18262

**Published:** 2024-03-23

**Authors:** Xiang Zhang, Qingqing Xiao, Cong Zhang, Qinghua Zhou, Tao Xu

**Affiliations:** ^1^ Lung cancer center, West China hospital Sichuan university Chengdu China; ^2^ Division of Abdominal Tumor Multimodality Treatment, Cancer Center, West China Hospital Sichuan University Chengdu China; ^3^ Department of Thoracic surgery Chengdu Seventh People's Hospital (Affiliated Cancer Hospital of Chengdu Medical College) Chengdu China; ^4^ Department of Thoracic Surgery The Affiliated Hospital, Southwest Medical University Luzhou China

**Keywords:** cancer‐associated fibroblasts, immunotherapeutic response, lung squamous cell carcinoma, prognostic signature, single‐cell RNA sequencing

## Abstract

Lung squamous cell carcinoma (LUSC) is one of the subtypes of lung cancer (LC) that contributes to approximately 25%–30% of its prevalence. Cancer‐associated fibroblasts (CAFs) are key cellular components of the TME, and the large number of CAFs in tumour tissues creates a favourable environment for tumour development. However, the function of CAFs in the LUSC is complex and uncertain. First, we processed the scRNA‐seq data and classified distinct types of CAFs. We also identified prognostic CAFRGs using univariate Cox analysis and conducted survival analysis. Additionally, we assessed immune cell infiltration in CAF clusters using ssGSEA. We developed a model with a significant prognostic correlation and verified the prognostic model. Furthermore, we explored the immune landscape of LUSC and further investigated the correlation between malignant features and LUSC. We identified CAFs and classified them into three categories: iCAFs, mCAFs and apCAFs. The survival analysis showed a significant correlation between apCAFs and iCAFs and LUSC patient prognosis. Kaplan–Meier analysis showed that patients in CAF cluster C showed a better survival probability compared to clusters A and B. In addition, we identified nine significant prognostic CAFRGs (CLDN1, TMX4, ALPL, PTX3, BHLHE40, TNFRSF12A, VKORC1, CST3 and ADD3) and subsequently employed multivariate Cox analysis to develop a signature and validate the model. Lastly, the correlation between CAFRG and malignant features indicates the potential role of CAFRG in promoting tumour angiogenesis, EMT and cell cycle alterations. We constructed a CAF prognostic signature for identifying potential prognostic CAFRGs and predicting the prognosis and immunotherapeutic response for LUSC. Our study may provide a more accurate prognostic assessment and immunotherapy targeting strategies for LUSC.

## 1 INTRODUCTION

Lung cancer (LC) is one of the most damaging malignancies and one of the major causes of cancer‐related deaths worldwide.^1^ Lung squamous cell carcinoma (LUSC) is one of the subtypes of non‐small cell lung cancer, representing about 25%–30% of all LC and making it the 2nd most common LC type.^2^, ^3^ The majority of LUSC originates in the larger bronchi and often displays central lung cancer. LUSC grows rapidly and has a much higher incidence rate in males than in females.^4^ In addition, long‐term smoking is considered a strong correlate for the development of LUSC.^5^ LUSC causes enormous strain on society's healthcare system owing to its high incidence, high mutation rate, low survival rate and high recurrence rate.^6^ Due to the lack of specific symptoms in early LUSC, most LUSC patients are in the intermediate or advanced stage when they are diagnosed and have poor treatment outcomes.^7^ Although surgical resection is currently the most efficient way to eradicate LC in clinical settings, it might not be appropriate for every patient. Despite the breakthroughs in immunotherapy and targeted therapy in the treatment of LUSC, LUSC patients still have a worse outcome and poor prognosis due to tumour heterogeneity and drug resistance.^8^, ^9^ Therefore, exploring the underlying pathological mechanisms and identifying novel therapeutic targets have become the focus of current research on LUSC.

Cancer‐associated fibroblasts (CAFs) are a specific type of fibroblast that were identified in the tumour mesenchyme of multiple cancers, such as LUSC.^10^ CAFs are key cellular components of the tumour microenvironment (TME), and the large number of CAFs in tumour tissues builds a favourable environment for tumour development.^11^ CAF regulates malignant biological behaviours of tumour cells, including proliferation, metastasis and drug resistance, through the secretion of special cytokines and extracellular matrix (ECM).^12^ Additionally, CAFs could promote angiogenesis and regulate T‐cell activity, leading to immunosuppression of tumours.^13^ Numerous studies have confirmed that cancer‐associated fibroblast‐related genes (CAFRGs) can be used as specific biomarkers for prognostic prediction in cancer, including bladder cancer,^14^ colorectal cancer,^15^ and colon cancer.^16^ While the function of CAFs in the LUSC is complex and uncertain, screening for CAFRGs in LUSC is necessary to obtain a fuller view of the contribution of CAFs to the TME in LUSC.

Previously, the lack of knowledge about the cellular subpopulations of CAFs and TME heterogeneity made the identification of specific effector subpopulations more difficult, which consequently hampered the detection of specific biomarkers and genes, resulting in poor clinical targeting of tumours.^17^ The scRNA‐seq technology can more accurately investigate the heterogeneity of tumour cells and the changes in the immune landscape of TME, providing more reliable basis evidence for a deeper understanding of the pathogenesis of tumours, along with treatment strategies and prognosis.^18^, ^19^ A scRNA‐seq study of oesophageal squamous cell carcinoma revealed that malignant epithelial cells exhibit a highly heterogeneous state and are enriched in the oxidative phosphorylation pathway, which provided the basis for a deeper understanding of the pathogenesis and progression of oesophageal squamous cell carcinoma.^20^ Another scRNA‐seq result showed that 21 vital tumour mutant genes were identified in bladder cancer samples, with mutations in ARID1A, GPRC5A and MLL2 increasing the self‐renewal capacity of tumour cells and contributing to tumorigenesis.^21^ Previous research adopted scRNA‐seq technology to classify and identify T lymphocyte subpopulations in the peripheral blood, cancer tissue and adjacent tissue of non‐small cell lung cancer patients while analysing the expression of drug target genes. The study might offer new potential targets for the development of immune therapy methods that specifically target T‐cell subpopulations.^22^ The scRNA‐seq technology is a great breakthrough in oncobiology research, achieving comprehensive and intuitive understanding of malignant tumours from multiple perspectives and levels and thus guiding clinical diagnosis, treatment and prognosis.

First, we processed the scRNA‐seq data. Then, we recognized fibroblasts and classified three distinct types of CAFs to recognize highly variable genes. Besides, we screened prognostic CAFRGs by univariate Cox analysis and performed a survival analysis to test the effect of three different CAF subtypes on the prognosis for LUSC. We conducted a consensus clustering analysis to investigate the correlation between different CAF clusters and clinical features. In addition, we used the ssGSEA to quantitatively assess the immune landscape in different CAF clusters. We developed a model with a significant prognostic correlation and verified the prognostic model. The enrichment and gene mutation analyses were used to elucidate the underlying molecular mechanisms contributing to the variability in LUSC risk. Furthermore, we explored the immune landscape of LUSC and further investigated the correlation between malignant features and LUSC. Our study might provide a more accurate prognostic assessment and effective treatment strategies for LUSC by identifying potential prognostic CAFRGs and immunotherapeutic targets.

## 2 MATERIALS AND METHODS

### 2.1 Downloading of data

The scRNA‐seq data for LUSC were gained from the GEO database, more specifically GEO‐GSE153935 (https://www.ncbi.nlm.nih.gov/geo/query/acc.cgi?acc=GSE153935), which comprised 8158 single‐cells derived from 18 LUSC samples. The RNA‐seq data were obtained from multiple sources, namely TCGA, including TCGA‐LUSC and TCGA‐LUAD (https://portal.gdc.cancer.gov/), as well as from GEO repositories, including GEO‐GSE74777 (https://www.ncbi.nlm.nih.gov/geo/query/acc.cgi?acc=GSE74777) and GEO‐GSE157010 (https://www.ncbi.nlm.nih.gov/geo/query/acc.cgi?acc=GSE157010).^23^, ^24^ After carefully discarding incomplete and duplicate data, the TCGA‐LUSC dataset consisted of 473 samples, the TCGA‐LUAD dataset included 450 samples, the GEO‐GSE74777 dataset had 107 samples and the GEO‐GSE157010 dataset retained 234 samples. Additionally, RNA‐seq and relevant clinical data were obtained from a total of 8739 samples across 32 different types of tumours from the UCSC Xena portal (https://xenabrowser.net/datapages/).^25^ The open‐access database used in this study does not require additional ethical approval. Our data collection and use procedures are carried out in accordance with relevant regulations.

### 2.2 Processing of scRNA‐seq data

We employed the ‘Seurat’ R package to create a Seurat object with the objective of scrutinizing the scRNA‐seq data.^26^ Subsequently, we retained cells that exhibited gene numbers greater than 300 or less than 10,000, UMI counts exceeding 600, haemoglobin percentages lower than 1% and mitochondrial gene percentages below 20%. This filtering process resulted in a final collection of 8049 cells. Additionally, we tackled potential cell cycle effects, performed data normalization, performed dimensionality reduction from 1 to 30, applied clustering analysis with a resolution of 1 and assigned cell annotations to the Seurat object.^27^, ^28^

Subsequent to the foregoing, we identified fibroblasts and proceeded to undertake a similar analysis as before. Specifically, we classified three distinct types of CAFs, namely inflammatory CAFs (iCAFs), myoblastic CAFs (mCAFs) and antigen‐presenting CAFs (apCAFs), by utilizing the ‘FindAllMarkers’ function in order to recognize highly variable genes (log2FC ≥0.3, min.pct = 0.1 and diff.pct ≥0.1). We employed the ‘Monocle 2’ package to analyse the different differentiation states of CAF subpopulations.^29^ In addition, we implemented ‘pySCENIC’ to assess the level of transcription factor activation in various CAF subtypes.^30^ Finally, we analysed communication patterns based on ligand‐receptor information using ‘CellChat’, which modelled communication probability and successfully identified significant communications.^31^

### 2.3 Consensus clustering analysis

We conducted a survival analysis to examine the effect of CAF subtypes on the prognosis for LUSC. Initially, we performed a univariate Cox analysis to evaluate the prognostic significance of CAFRGs (*p*‐value <0.05). Additionally, we employed the package ‘ConsensusClusterPlus’ to perform a consensus unsupervised clustering analysis, with 100 repetitions, using the *k*‐means clustering algorithm and Euclidean distance metric.^32^ We then explored the differences in survival among various CAF clusters. Moreover, we utilized principal component analysis (PCA), t‐distributed stochastic neighbour embedding (tSNE) and uniform manifold approximation and projection (UMAP) analysis to find the distribution of CAF clusters.^33^ We further defined the expression patterns of prognostic CAFRGs in CAF clusters to recognize unique CAF regulatory patterns.^34^ Finally, using ‘pheatmap’, we examined the relationships between CAF clusters and clinical profiles to recognize the potential biological mechanisms for different clinical and pathological features in LUSC samples.

### 2.4 Immune cell infiltration and enrichment analyses

Initially, we used the ssGSEA to evaluate the immune landscape in different CAF clusters.^35^ This analysis enabled us to determine both the abundance and activity of immune cell populations in each cluster, subsequently identifying significant differences by the Wilcoxon test. Following this, we conducted the GSVA method to assess the activity status of KEGG pathways in each CAF cluster, offering a generalized appraisal of the overall pathway activity within each group.^35^ The top 30 enriched pathways were visualized via a heatmap, offering an overview of pathway activation patterns among the various clusters. This allowed us to identify the key signalling pathways that were dysregulated in each CAF cluster.

### 2.5 Development of prognostic model

To improve the accuracy of our prognostic signature, we developed a signature with significant prognostic CAFRGs. To accomplish this, we randomly divided our dataset into training and test sets utilizing the ‘caret’ R package, following a 7:3 ratio. Subsequently, we narrowed down the most valuable CAFRGs by performing LASSO analysis with 10‐fold cross‐validation. Finally, we employed a multivariate Cox analysis to develop a prognostic model based on the identified CAFRGs. Individual sample risk scores were obtained using the formula:

$$\text{Risk score}=\sum_{i=1}^{k}\text{βiSi}$$

### 2.6 Validation of prognostic signature

To assess the differences in survival rates among risk groups in our model, we utilized Kaplan–Meier analysis with a chi‐squared test. Furthermore, we validated the model's stability by employing the test set, whole set, TCGA‐LUAD, GEO‐GSE74777 and GEO‐GSE157010 sets. We evaluated the predictive performance of both our model and clinical features using ROC curves, which were analysed via the ‘timeROC’ R package.^36^ Additionally, we utilized the HPA database to examine the protein expression levels of the prognostic CAFRGs. Moreover, we delved into the CNV landscape associated with these prognostic CAFRGs to identify any genomic alterations that may influence gene expression patterns.^37^, ^38^

Furthermore, we explored the interrelationship between these prognostic CAFRGs to uncover potential co‐expression patterns indicative of functional associations among these genes.^39^, ^40^ The C‐index was employed to measure the discriminative capacity of the signature compared to clinical characteristics. For convenience of clinical implementation, a nomogram combining the model and clinical characteristics was formulated to forecast the survival probabilities.

### 2.7 Enrichment and gene mutation analyses

The objective of the research was to illuminate the potential molecular mechanisms contributing to the variability in LUSC risk. To achieve this, we employed advanced techniques to identify differentially expressed genes (DEGs) among distinct risk‐level groups with high‐stringency criteria (|log2FC| ≥ 1 and FDR <0.05). These significant DEGs were then subjected to GO and KEGG analyses (*p*‐value <0.05) to explore the key molecular pathways involved in LUSC pathogenesis.^41^ Additionally, we utilized the ‘maftools’ package to analyse and visualize somatic mutations across different risk groups.^42^

### 2.8 Exploration of the immune landscape

To explore the TME of LUSC, we utilized several sophisticated methods to assess immune cell infiltration.^43^, ^44^, ^45^, ^46^, ^47^, ^48^, ^49^, ^50^, ^51^ We evaluated immune function in risk groups by employing the GSVA and comparing scores across risk groups using the Wilcoxon test. We investigated the differences in immune checkpoint gene (ICG) expression levels between various groups by the Wilcoxon test. Additionally, we calculated the TIDE scores for each risk group to predict the response to immunotherapy and conducted a comparative analysis.^52^

### 2.9 Identification of drugs

To investigate the potential efficacy of different medications against LUSC, we employed the ‘oncoPredict’ package to predict drug responsiveness using the GDSC database.^53^ Using this approach, our study successfully identified potential effective medications against LUSC by evaluating the GDSC database. The neural network‐based prognostic model utilized in this study considers the genomic and transcriptomic features of cancer cell lines, thus enabling the identification of potential drug targets for the treatment of LUSC. These findings pave the way for the development of novel treatment strategies that target the specific genomic attributes of LUSC, thus offering potential avenues for personalized medical approaches to managing this disease.

### 2.10 Correlation with malignant features

In addition, we performed the z‐score technique to achieve a more precise representation of specific biological pathways.^25^, ^54^ The analysis focused on gene sets related to crucial biological processes, including angiogenesis, cell cycle regulation, EMT and CAFRGs. The recently established GSVA approach, implemented through the package ‘GSVA’, was used.^55^, ^56^

### 2.11 Statistical Analyses

The statistical analyses for this study were performed using R software version 4.3.0. In order to compare and evaluate the expressions between the different groups, the Wilcoxon test was employed. The statistical significance threshold was set at *p* < 0.05.

## 3 RESULTS

### 3.1 Identification of different CAFs

**FIGURE 1:**
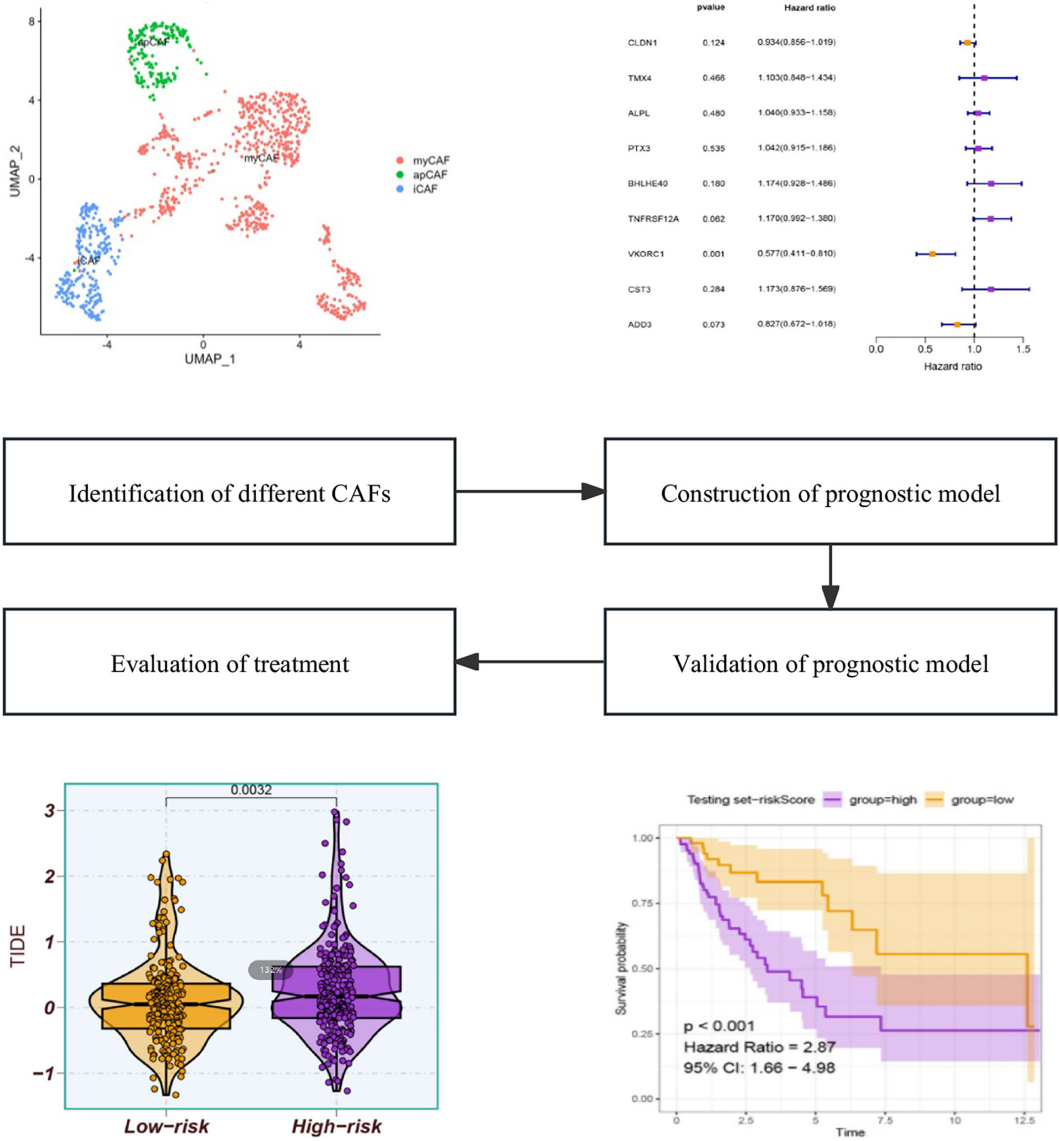
The overall workflow.

The study is depicted in Figure 1, outlining the overall workflow. The study procured 18 LUSC samples and 8049 cells from the GSE153935 dataset following rigorous quality control (Figure S1A). The package ‘clustree’ visually represented the interactions between various clusters at differing resolutions, aiding in the selection of a suitable resolution for downstream analysis (Figure S1B). Cell cycle effect removal, normalization, dimensionality reduction and clustering were performed, and distinct clusters were represented via the UMAP plot (Figure S1C). The subsequent cell annotations were performed for each of these clusters (Figure S1D). Additionally, bubble plots were utilized to visualize marker gene expression levels for each cell type (Figure S1E). Upon the extraction of fibroblasts, further investigations were carried out, and the distribution of eight distinct clusters through the UMAP plot was obtained (Figure S2A–C). Cell annotations on every CAF were performed (Figure S2D). The ‘FindAllMarkers’ function recognized 1000 genes for each high variant gene (log2FC ≥0.3, min.pct = 0.1 and diff.pct ≥0.1) (Table S1). Furthermore, cell differentiation trajectories for distinct CAFs were constructed via Monocle 2, based on pseudotimes, cell types and different samples (Figure S3A). Additionally, the varying transcription factor activation based on distinct CAFs is illustrated in Figure S3B. The analysis also explored the interactions between CAFs and other cells, wherein CAFs exerted a more pronounced effect on other cells (Figure S4).

### 3.2 Classification of CAF clusters

**FIGURE 2:**
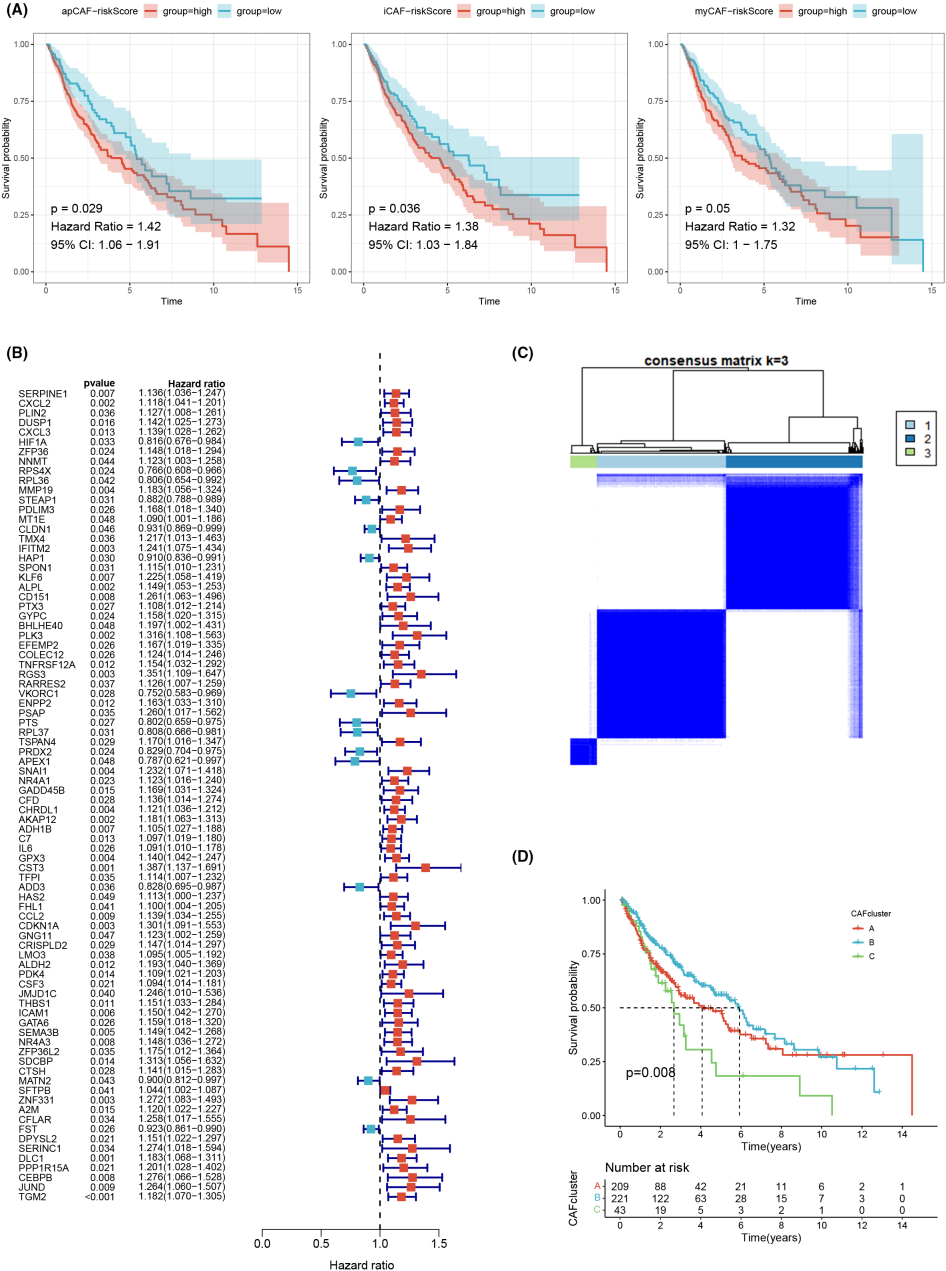
(A) A significant correlation between apCAFs and iCAFs with patient prognosis. (B) 84 CAFRGs were identified by univariate Cox analysis. (C) Consensus matrix at optimal *k* = 3. (D) Patients in CAF cluster C exhibited significantly better survival probabilities compared to those in clusters A and B.

To investigate the prognostic significance of the distinct CAFs for LUSC, a survival analysis was conducted. The results indicated a significant correlation between apCAFs and iCAFs and patient prognosis (Figure 2A). Furthermore, a univariate Cox analysis was performed to recognize prognostic CAFRGs, resulting in the identification of 84 CAFRGs with prognostic potential (Figure 2B). In order to classify LUSC patients in the 84 CAFRGs, the consensus clustering method was employed. The optimal number of clusters (*K*) was determined based on criteria. Based on these criteria, *K* = 3 was chosen as the most suitable number of clusters. The whole set was then divided into three distinct CAFRG clusters, labelled as clusters A, B and C (Figure 2C and Figure S5). Additionally, Kaplan–Meier curve analysis revealed that patients in CAF cluster C showed a better survival probability compared to those in clusters A and B (Figure 2D).

**FIGURE 3:**
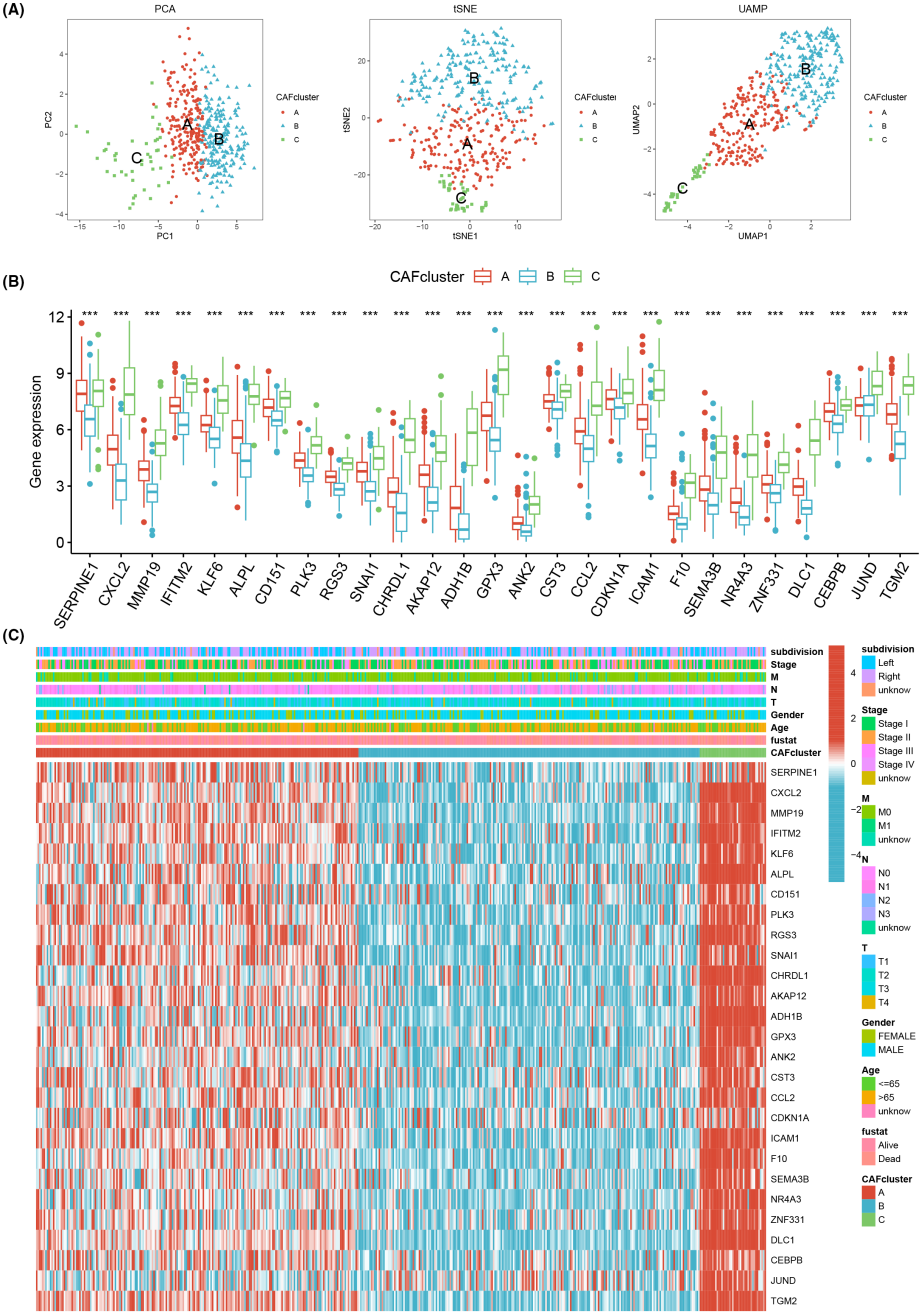
(A) The PCA, tSNE and UMAP algorithms indicated significant distinctions among the three subtypes. (B) Higher expression levels of CAFRG in CAF cluster C. (C) The correlations between the two CAFRG clusters and various clinicopathological features, including TNM stage, gender, age, survival time and survival status.

**FIGURE 4:**
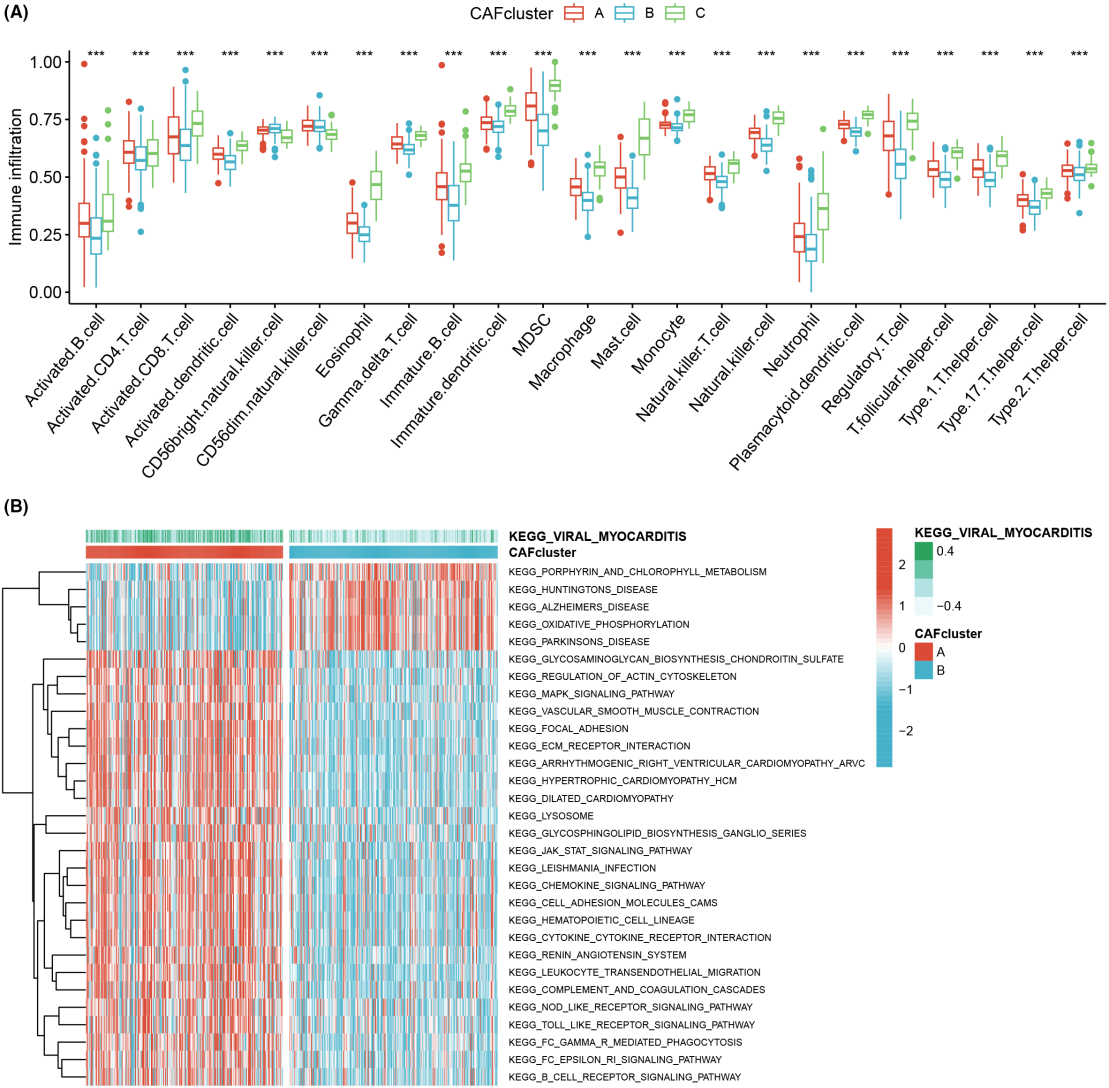
(A) A notable increase in immune cell infiltration in CAF cluster C. (B) Distinct molecular pathways across the three CAF clusters.

The findings from the PCA, tSNE and UMAP algorithms provided further evidence of the significant distinctions among the three subtypes, thereby validating the robustness of the identified subtypes (Figure 3A). Analysis of gene expression patterns revealed higher expression levels of CAFRG in CAF cluster C, suggesting a greater abundance of CAFs in samples belonging to this cluster (Figure 3B). Moreover, we investigated the correlations between the two CAFRG clusters and various clinicopathological features, including TNM stage, gender, age, survival time and survival status (Figure 3C). In order to explore patterns of immune cell infiltration among the different CAF clusters, ssGSEA was performed. The results demonstrated a notable increase in immune cell infiltration in CAF cluster C. For example, compared with CAF clusters A and B, activated B cells, immature B cells, mast cells, neutrophils, NK cells and eosinophils were significantly highly expressed in CAF cluster C, suggesting that CAF cluster C may be more sensitive to immunotherapy (Figure 4A). Additionally, the results of GSVA indicated distinct molecular pathways across the three CAF clusters (Figure 4B and Figure S6).

### 3.3 Construction and validation of prognostic model

**FIGURE 5:**
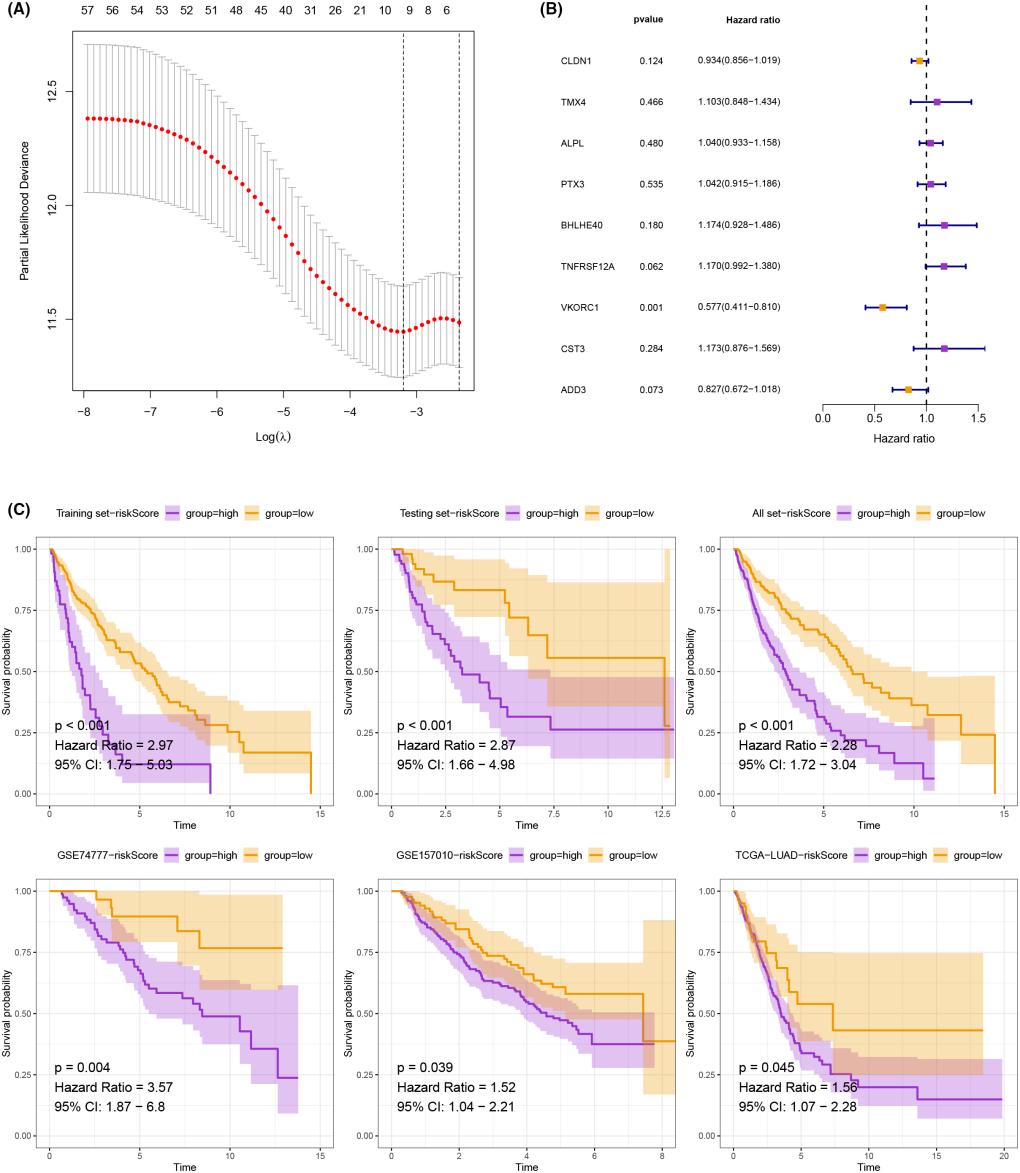
(A) 9 significant CAFRGs were identified by LASSO analysis. (B) A model comprising 9 CAFRGs was constructed by multivariate Cox analysis. (C) Patients with LUSC belonging to the low‐risk group exhibited remarkably higher survival rates across multiple internal (train, test and full) and external sets (comprising TCGA‐LUAD, GEO‐GSE74777 and GEO‐GSE157010 sets).

The entire set of samples related to LUSC was randomly partitioned into two groups, namely a train set and a test set, to achieve a ratio of 7:3. In order to identify prognostic CAFRGs, we utilized LASSO analysis, which revealed nine significant CAFRGs (Figure 5A), and subsequently employed multivariate Cox analysis to formulate a model comprising nine CAFRGs (Figure 5B). Patients with LUSC belonging to the low‐risk group exhibited remarkably higher survival rates across multiple internal (train, test and full) and external sets (comprising TCGA‐LUAD, GEO‐GSE74777 and GEO‐GSE157010 sets) (Figure 5C). These outcomes provide strong evidence supporting the prognostic potential of the model for LUSC patients. Moreover, the AUC for the model at 5 years was greater than 0.7, surpassing that of the clinical features and indicating high reliability (Figure S7).

Furthermore, the present study investigated the protein expression levels of LUSC tumour and normal tissues by employing the HPA database. Notably, TMX4, ALPL, PTX3, BHLHE40, TNFRSF12A and CST3 demonstrated significant overexpression in LUSC tumour tissues, whereas CLDN1, VKORC1 and ADD3 exhibited significant underexpression (Figure S8). Additionally, to determine the specific influence of mutations on CAFRGs, the study pinpointed the chromosomal locations of CNVs (Figure S9A). The CAF network analysis provided an understanding of the interconnections and regulatory linkages among CAFRGs (Figure S9B). Furthermore, the study scrutinized the CNV patterns of the nine prognostic CAFRGs, revealing that genes such as CLDN1, PTX3, VKORC1, TMX4, BHLHE40 and CST3 exhibited a higher frequency of CNV amplification, while others, including ADD3, TNFRSF12A and ALPL, demonstrated a higher frequency of missing CNVs, thereby implying deletions (Figure S9C).

**FIGURE 6:**
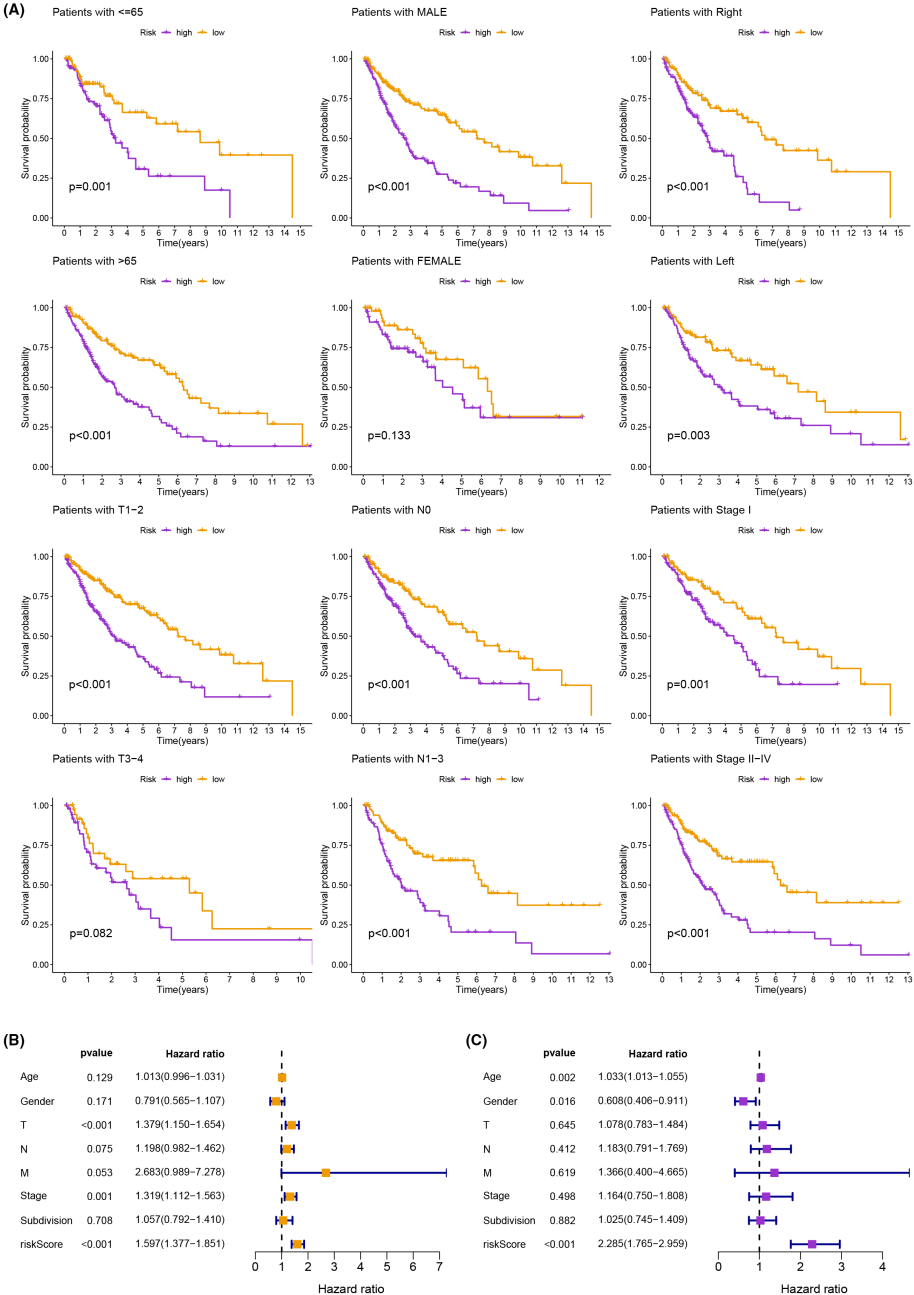
(A) Consistently favourable survival outcomes were observed in patients classified as lower‐risk, between different clinical subgroups. (B, C) Both univariate and multivariate Cox analyses confirmed the independent prognostic ability of the model in predicting the prognosis of LUSC patients.

**FIGURE 7:**
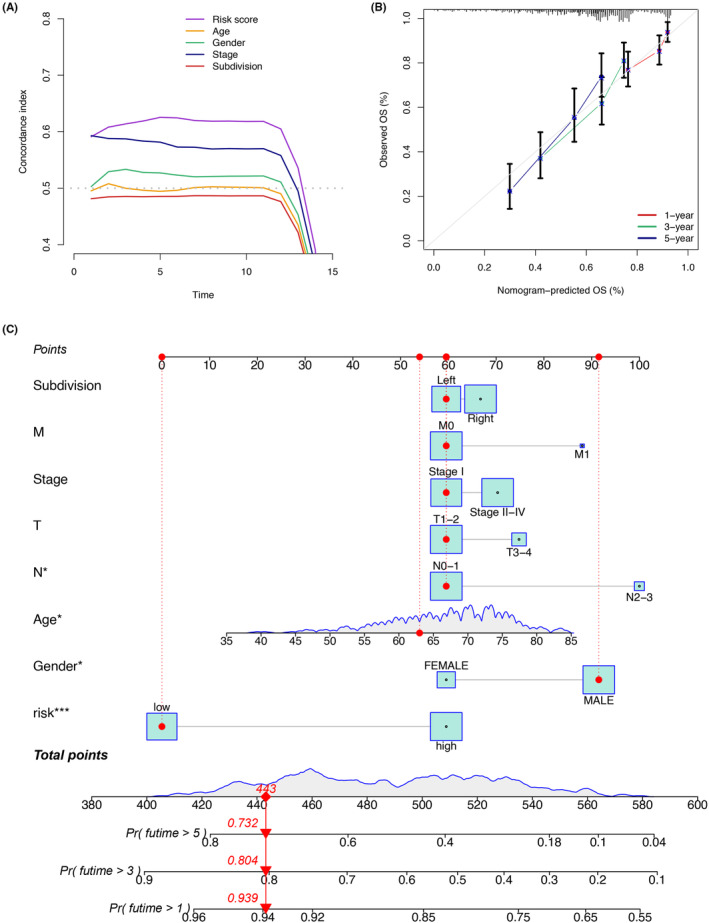
(A) The C‐index analysis indicated the superior prognostic efficacy of the signature in comparison to clinical characteristics. (B, C) A nomogram was developed, integrating the model with clinical characteristics to predict the survival probabilities of LUSC patients at 1, 3 and 5 years.

Additionally, we intended to assess the generalizability and practicality of the developed prognostic signature across different patient populations. Intriguingly, consistently favourable survival outcomes were observed in patients classified as lower‐risk across clinical subgroups, thereby highlighting the usefulness and adaptability of the signature (Figure 6A). Both univariate and multivariate Cox analyses further confirmed the independent prognostic ability of the model in predicting the prognosis of LUSC (Figure 6B,C). In addition, the C‐index analysis indicated the superior prognostic efficacy of the signature in comparison to clinical characteristics (Figure 7A). To enable the practical application of the model in a clinical environment, a nomogram was developed, integrating the model with clinical characteristic to predict the survival probabilities of LUSC patients. Remarkably, it was observed that the different groups had the most substantial impact on prognosis (Figure 7B,C).

### 3.4 Enrichment and mutation analysis

We identified 258 DEGs among different groups (|log2FC ≥1| and FDR <0.05 and Table S2). To gain a comprehensive understanding of the biological functions and molecular pathways of DEGs, we performed GO and KEGG analyses. The GO analysis revealed that the BP terms of these DEGs were primarily associated with humoral immune response, antibacterial humoral response and antimicrobial humoral response. The CC terms were associated with the lamellar body, platelet alpha granule lumen and secretory granule lumen. On the other hand, the MF terms were predominantly associated with receptor ligand activity, signalling receptor activator activity and cargo receptor activity (Figure S10A and Table S3). KEGG pathway analysis presented various pathways that were enriched in DEGs, including IL‐17 signalling pathway, complement and coagulation cascades, haematopoietic cell lineage, amoebiasis, pertussis and staphylococcus aureus infection (Figure S10B and Table S4). Furthermore, Figure S10C,D depicts no significant differences in gene mutations among different risk groups.

### 3.5 Exploration of the immune landscape

**FIGURE 8:**
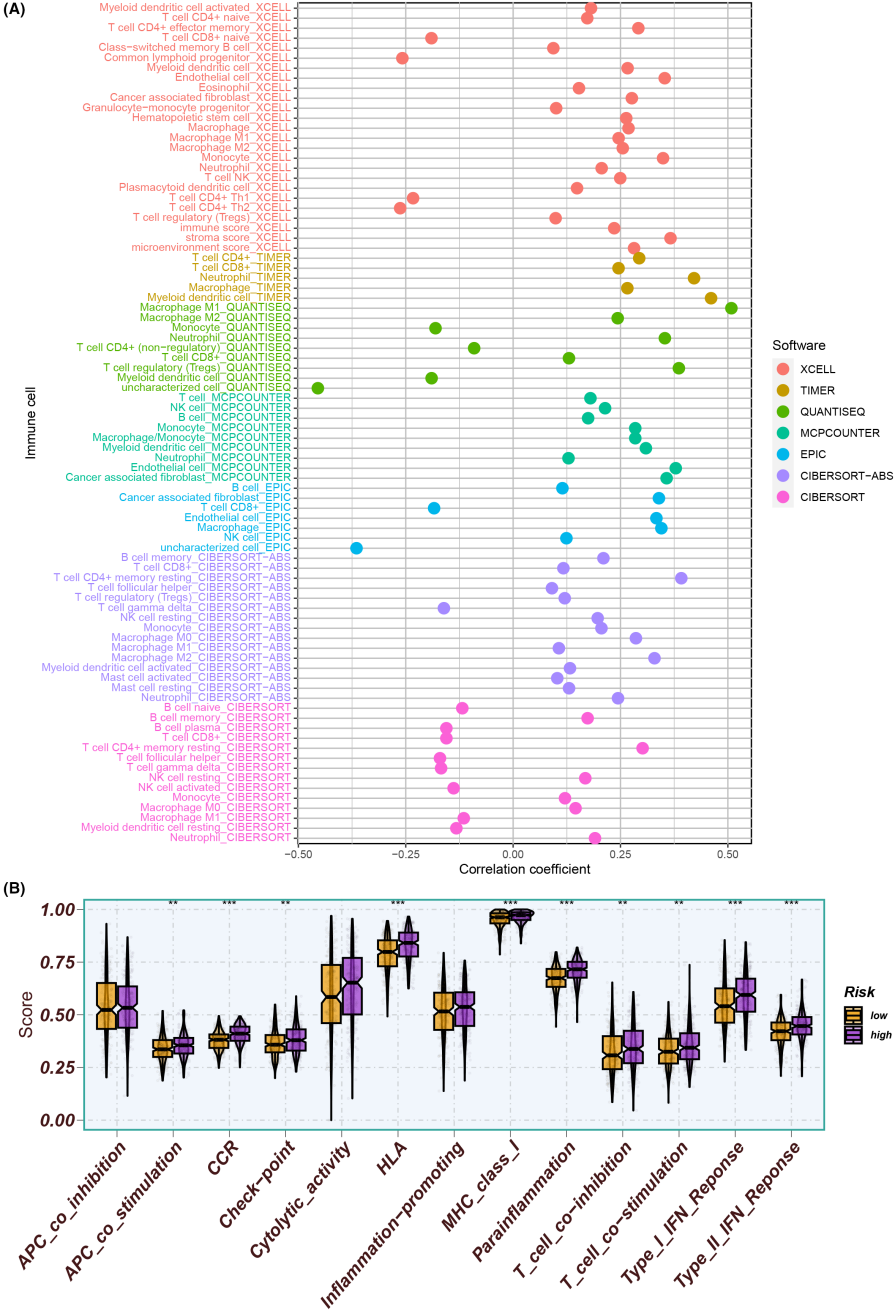
(A) A positive association between risk scores and the majority of immune cell subpopulations. (B) In addition to APC co‐inhibition, cytolytic activity and inflammation‐promoting, higher levels of immune function scores were observed in the high‐risk group.

**FIGURE 9:**
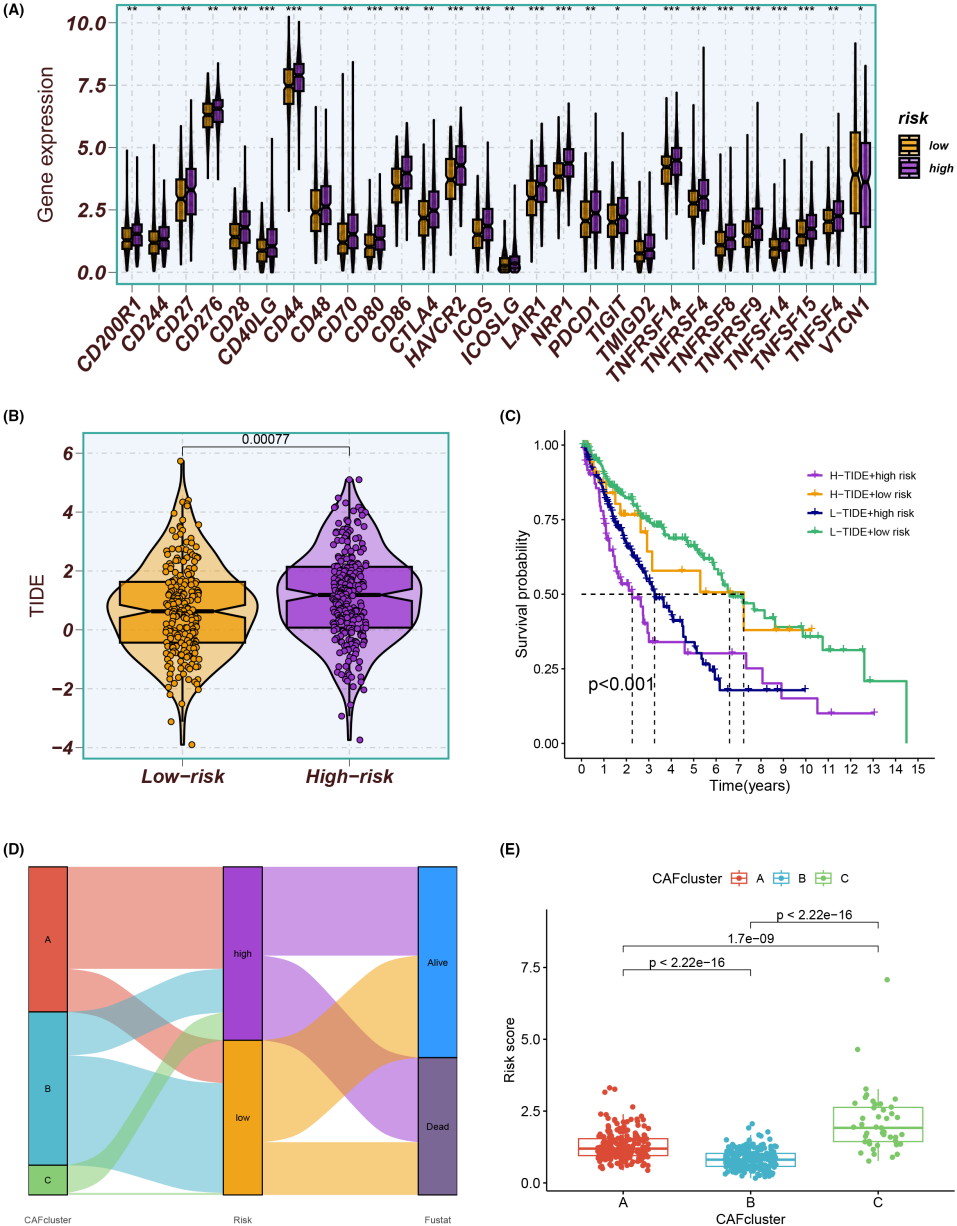
(A) The high‐risk group exhibited significant upregulation of several ICGs. (B) The low‐risk group exhibited lower TIDE scores, indicating a potential responsiveness to immunotherapeutic interventions. (C) The combination of TIDE scores and risk scores was demonstrated to be a robust predictor of patient prognosis. (D, E) The Sankey plot provided informative insights into the associations between CAF clusters, risk groups and survival outcomes. CAF cluster A was linked to higher risk scores.

The results indicated a positive correlation with risk scores and the majority of immune cell subpopulations, suggesting higher levels of immune cell infiltration in the high‐risk group (Figure 8A). In addition to APC co‐inhibition, cytolytic activity and inflammation‐promoting, higher levels of immune function scores were observed in the high‐risk group (Figure 8B). Moreover, the high‐risk group exhibited significant upregulation of several ICGs (Figure 9A). Conversely, the low‐risk group exhibited lower TIDE scores, indicating a potential responsiveness to immunotherapeutic interventions (Figure 9B). The combination of TIDE scores and risk scores was demonstrated to be a robust predictor of patient prognosis (Figure 9C). Furthermore, the Sankey plot provided informative insights into the associations between CAF clusters, risk groups and survival outcomes. Specifically, CAF cluster A was linked to higher risk scores (Figure 9D,E).

### 3.6 Identification of drugs

**FIGURE 10:**
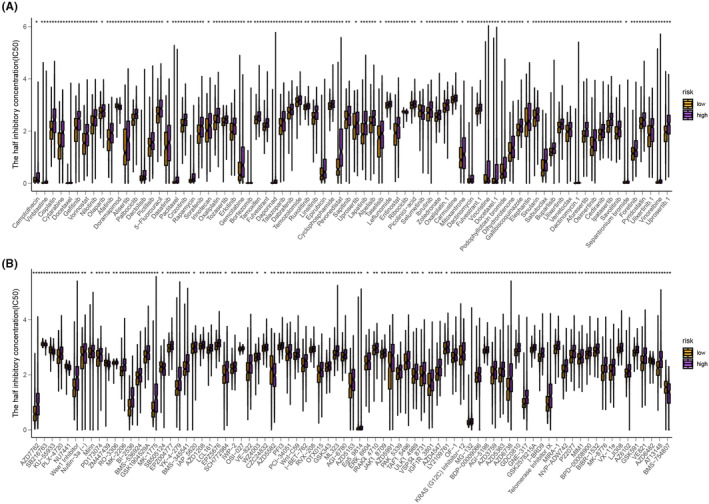
(A, B) This screening involved the examination of 78 chemotherapeutic drugs and 81 targeted therapeutic drugs (*p*‐value <0.05).

To identify potential effective drugs for LUSC, we performed a comprehensive drug screening using the ‘oncoPredict’ R package on the GDSC database. This screening involved the examination of 78 chemotherapeutic drugs and 81 targeted therapeutic drugs (*p*‐value < 0.05) (Figure 10A,B).

### 3.7 Correlation with malignant features

Tumour cells exhibit a diverse array of distinct characteristics that facilitate their uncontrolled growth, invasion, metastasis, angiogenesis and induction of EMT, eventually promoting tumour development. CAFRG is recognized as a key element in cancer progression and the development of drug resistance. The findings demonstrate a strong and statistically significant correlation between the CAFRG z‐score and the angiogenesis z‐score (*R* = 0.33, *p* < 0.001), EMT z‐score (*R* = 0.38, *p* < 0.001) and cell cycle z‐score (*R* = −0.21, *p* < 0.001) across the TCGA pan‐cancer cohort (Figure S11).

## 4 DISCUSSION

LUSC is a common histological subtype of LC and one of the major causes of cancer‐related deaths worldwide.^55^, ^56^ The main treatments for LUSC include surgery, radiotherapy, chemotherapy, targeted therapy and immunotherapy. Yet, LUSC‐specific clinical pathological features and the tendency for early metastasis make clinical treatment more challenging.^57^ Immune inhibitors combined with chemotherapy have become a preferred option for LUSC treatment in recent years, although satisfactory results have not been achieved owing to the heterogeneity of the tumours.^58^ Therefore, screening disease targets and mining new therapeutic approaches have become the focus of attention at the present stage. In the study, we identify biomarkers, explore potential immune targets and develop personalized treatment strategies to enable more patients to achieve better outcomes in cancer treatment.

The TME is the soil that tumours rely on for survival, consisting of different types of cells within the tumour, tumour blood vessels, secreted factors and ECM.^59^ The TME constantly interacts with tumour cells, thus influencing disease progression.^60^ Several studies have proven that TME acts critically in promoting tumour growth, such as drug resistance, immune escape, malignant metastasis and immunosuppression.^61^, ^62^ In TME, CAFs supported tumour cell growth and multiplication by constructing a highly inflammatory, immunosuppressive and vascular growth environment.^63^ CAFs could promote the occurrence, development and invasive metastasis of LUSC.^64^ High levels of CAFs are closely related to a poor prognosis in LUSC patients.^10^ Based on the changes in TME and CAFs, multiple immunotherapies for cancer were developed and applied in the clinic. Nevertheless, the heterogeneity and complexity of the tumour made the actual clinical outcome and the prognosis worse for cancer patients.^65^ Hence, further exploring and exploitation of the features of TME and CAFs is crucial for enhancing treatment efficacy and improving prognosis in cancer patients.

First, we gained the scRNA‐seq data and RNA‐seq data from public datasets, which were filtered for further analysis. And the ‘Seurat’ package was used to scrutinize the scRNA‐seq data, resulting in 18 LUSC samples and 8049 cells from the GSE153935 dataset. Then we identified CAFs and classified them into three categories: iCAFs, mCAFs and apCAFs. ICAFs are a subtype of CAFs with a hyperinflammatory profile of α‐SMA^low^IL‐6^high^ and deficient myofibroblast profile.^66^ ICAFs were mediated by paracrine factors released by tumour cells. Notably, iCAFs exhibited spatial separation from both the tumour cells and myCAFs.^67^ MyCAFs, a subtype of CAFs with α‐SMA^high^ IL‐6^low^ myofibroblastic features, are activated when tumour cells come into direct contact with pancreatic stellate cells. ApCAFs is a CAFs subtype that expresses MHC II family genes with antigen processing and presentation functions.^68^ Another study found that apCAFs highly expressed H2‐Ab1, CD74 and serum amyloid A3.^69^ However, there is a need for further investigations aimed at elucidating the activation stimuli and spatial distribution of apCAFs. Further survival analysis showed that apCAFs and iCAFs were significantly associated with patient prognosis. The univariate Cox analysis was conducted to screen prognostic CAFRGs, which led to the identification of 84 CAFRGs with prognostic potential. We adopted the consensus clustering method to divide the entire cohort into three distinct CAFRG clusters, labelled clusters A, B and C. Besides, Kaplan–Meier analysis showed that patients in CAF cluster C showed a better survival probability compared to clusters A and B. The higher expression level of CAFRG in CAF cluster C indicates that there are more CAFs in the samples belonging to this cluster. The ssGSEA results showed that compared with CAFclusters A and B, activated B cells, immature B cells, mast cells, neutrophils, NK cells and eosinophils were significantly highly expressed in CAFcluster C, suggesting that CAFcluster C may be more sensitive to immunotherapy. B cell populations show significant heterogeneity in their surface immune phenotype and function.^70^ NK cells are the third group of lymphocytes alongside T and B cells and have wide spectrum anti‐tumour effects.^71^ Studies have shown that NK cells provide a significant immune barrier to LC growth and progression, and changing the state of NK cell activation may help control the disease process in LC.^72^ Mast cells could modulate the function of other immune cells in the TME, thereby affecting local immunosuppression or anti‐tumour immunity.^72^ And GSVA suggested distinct molecular pathways across the three CAF clusters.

Additionally, we utilized LASSO analysis to identify nine significant prognostic CAFRGs (CLDN1, TMX4, ALPL, PTX3, BHLHE40, TNFRSF12A, VKORC1, cST3 and ADD3) and subsequently performed multivariate Cox analysis to develop a prognostic model. CLDN1 (Claudin1) is a membrane protein that influences epithelial barrier function.^73^ CLDN1 is connected to the development of cancer, such as colon and breast cancer.^63^ Besides, CLDN1 targeting drug research achieved some investigation progress but is still in clinical research. ALPL is the alkaline phosphatase gene in the human body and performs vital physiological functions in tissues such as bone and liver.^74^ The latest study discovers ALPL‐1 as a potential target for osteosarcoma treatment.^75^ PTX3 is an important part of intrinsic immunity and serves a pivotal function in the fight against specific microorganisms and the regulation of inflammation.^76^ PTX3 levels were significantly higher in serum samples of PDAC patients, indicating that PTX3 may be a specific biomarker for pancreatic cancer.^77^ CST3 is a cysteine protease inhibitor and a biomarker for changes in kidney function.^78^ A breast cancer scRNA‐seq found CST3 overexpression in metastatic non‐TNBC cells.^79^ ADD3 is a cytoskeletal protein implicated in signal transduction, cell migration and adhesion. ADD3 is significantly downregulated in gliomas, demonstrating that its downregulation may promote tumour malignancy progress.^80^ Further validation analysis found that the low‐risk group of LUSC patients had higher survival in multiple internal and external sets. TMX4, ALPL, PTX3, BHLHE40, TNFRSF12A and CST3 demonstrated significant overexpression in LUSC tumour tissues, whereas CLDN1, VKORC1 and ADD3 exhibited significant underexpression in the HPA database. Furthermore, the immune landscape showed that risk scores were positively related to most immune cell subpopulations, indicating higher levels of immune cell infiltration in the high‐risk group. The high‐risk group exhibited significant upregulation in the ICG, such as CD28, ICOS, PDCD1 and NRP1. We also identify potential chemotherapeutic drugs and targeted therapeutic drugs for LUSC. Lastly, the correlation between CAFRG and malignant features indicates the critical utility of CAFRG in promoting tumour angiogenesis, EMT and cell cycle alterations, underscoring its therapeutic potential in cancer.

However, there were some shortcomings in the study. First, because we extracted data from public databases for our analyses, there might be an objective bias. Besides, because of the difficulty in collecting LUSC samples, we only used external datasets for validation, and no experimental validation was performed. To avoid potential biases, we validated the findings against external datasets to ensure the reliability and authenticity of our analyses. In addition, we have used various bioinformatics methods to deeply analyse and mine the prognosis, immune infiltration, TME and drug treatment response of LUSC from multi‐level and multi‐dimensional aspects, aiming to provide effective therapeutic strategies for the clinical treatment and prognosis of LUSC. In the future study, we will conduct further clinical and basic experiments to validate the results of the research.

## 5 CONCLUSION

We constructed a CAF prognostic signature model for identifying potential prognostic CAFRGs and predicting the prognosis and immunotherapeutic response in LUSC based on the scRNA‐seq data and bulk RNA‐seq data. Our study might provide a more precise prognostic appraisal and effective treatment strategies for LUSC by identifying immunotherapeutic targets.

## AUTHOR CONTRIBUTIONS

**Xiang Zhang:** Conceptualization (lead); writing – original draft (lead); writing – review and editing (lead). **Qingqing Xiao:** Conceptualization (equal); writing – original draft (lead); writing – review and editing (lead). **Cong Zhang:** Conceptualization (equal); data curation (equal); visualization (equal). **Qinghua Zhou:** Conceptualization (equal); writing – review and editing (equal). **Tao Xu:** Conceptualization (equal); project administration (equal); supervision (equal).

## FUNDING INFORMATION

No funding.

## CONFLICT OF INTEREST STATEMENT

The authors declare no conflict of interest. The authors declare that the research was conducted in the absence of any commercial or financial relationships that could be construed as a potential conflict of interest.

## Supporting information


Figures S1–S11

Tables S1–S4

## Data Availability

This study analysed data from TCGA and GEO databases. The processed data can be obtained from the corresponding author upon request.
