# Supplementary material for: Construction of a prognostic model with CAFs for predicting the prognosis and immunotherapeutic response of lung squamous cell carcinoma
Source: J Cell Mol Med. 2024 Mar 23;28(8):e18262. doi: 10.1111/jcmm.18262 (PMC10960179; doi:10.1111/jcmm.18262)
Supplement: Supplementary file 1 — Figures S1–S11 [file JCMM-28-e18262-s001.zip › Supplemental Legends.docx]

**SUPPLYMENTARY MATERIALS**

**Figure S1** (A) 18 LUSC samples and 8,049 cells from the GSE153935 dataset following rigorous quality control. (B) The interactions between various clusters at differing resolutions. (C) Distinct clusters were represented via the UMAP plot. (D) The cell annotations for each of these clusters. (E) Marker gene expression levels for each cell type.

**Figure S2** (A) The R package "clustree" visually represented the interactions between various clusters at differing resolutions. (B) Distinct clusters were represented via the UMAP plot. (C) The cell annotations for each of these clusters. (D) Cell annotations on CAFs.

**Figure S3** (A) Cell differentiation trajectories for distinct CAFs were constructed via Monocle 2, based on pseudotimes, cell types, and different samples. (B) the varying transcription factor activation based on distinct CAFs.

**Figure S4** (A-D) The interactions between CAFs and other cells.

**Figure S5** (A-G) Consensus matrices of the MRGs for k = 2, 4-9. (H and I) The cumulative distribution function plot and the consensus clustering matrix's delta area..

**Figure S6** (A and B) Distinct molecular pathways across the three CAF clusters.

**Figure S7** The AUC for the model at 5-years was greater than 0.7, surpassing that of the clinical features and indicating high reliability.

**Figure S8** Based on the HPA database, TMX4, ALPL, PTX3, BHLHE40, TNFRSF12A, and CST3 demonstrated significant overexpression in LUSC tumor tissues when compared to normal tissues, whereas CLDN1, VKORC1, and ADD3 exhibited significant underexpression.

**Figure S9** (A) The chromosomal locations of CNVs. (B) The interconnections and regulatory linkages among CAFRGs. (C) The CNV patterns of the nine prognostic CAFRGs reveal that genes such as CLDN1, PTX3, VKORC1, TMX4, BHLHE40, and CST3 exhibited a higher frequency of CNV amplification, while others, including ADD3, TNFRSF12A, and ALPL, demonstrated a higher frequency of missing CNVs, thereby implying deletions.

**Figure S10** (A and B) The GO and KEGG analyses for 258 DEGs. (B and C) No significant differences in gene mutations among different risk groups.

**Figure S11** A strong and statistically significant correlation between the CAFRG z-score and the angiogenesis z-score (R = 0.33, p < 0.001), EMT z-score (R = 0.38, p < 0.001), and cell cycle z-score (R = -0.21, p < 0.001) across the TCGA pan-cancer cohort.

**Table S1** 1000 high variant gene in every CAF.

**Table S2** 258 DEGs.

**Table S3** The GO analysis for 258 DEGs.

**Table S4** The KEGG analysis for 258 DEGs.
