# Supplementary figures and images for: Construction of a prognostic model with CAFs for predicting the prognosis and immunotherapeutic response of lung squamous cell carcinoma
Source: J Cell Mol Med. 2024 Mar 23;28(8):e18262. doi: 10.1111/jcmm.18262 (PMC10960179; doi:10.1111/jcmm.18262)

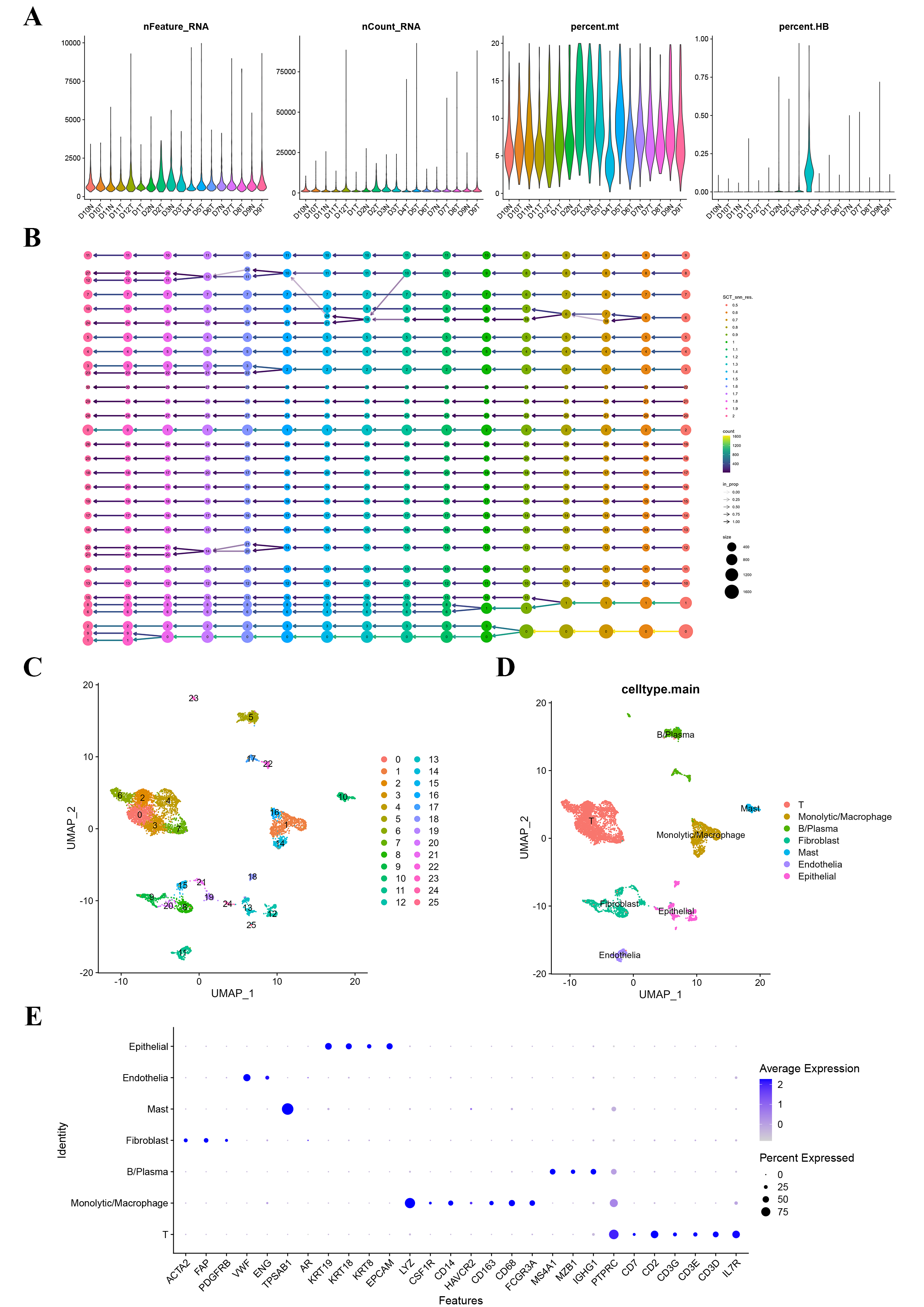

Supplement: Supplementary file 1 — Figures S1–S11 [file JCMM-28-e18262-s001.zip › Figure S1.tif]

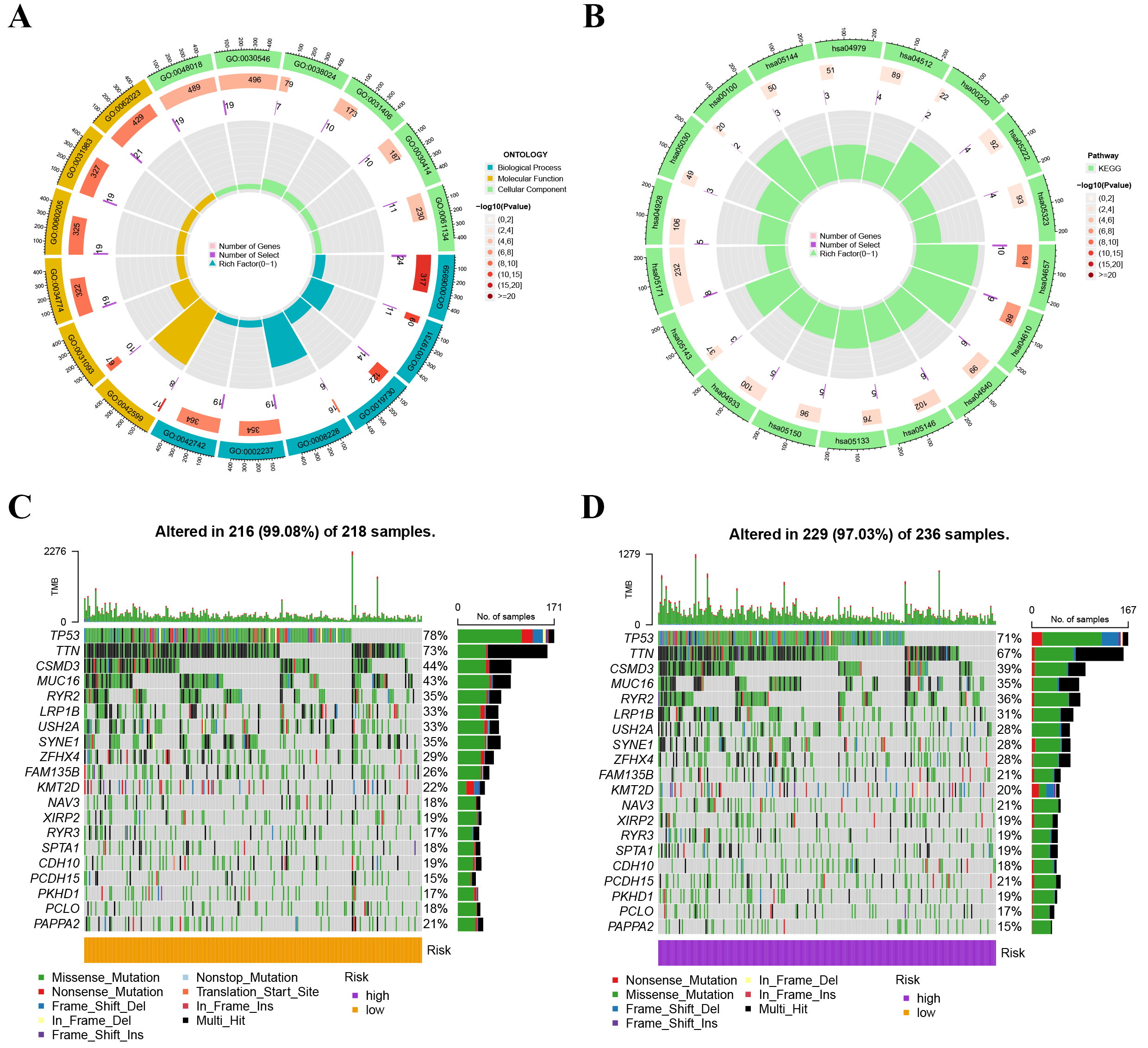

Supplement: Supplementary file 1 — Figures S1–S11 [file JCMM-28-e18262-s001.zip › Figure S10.tif]

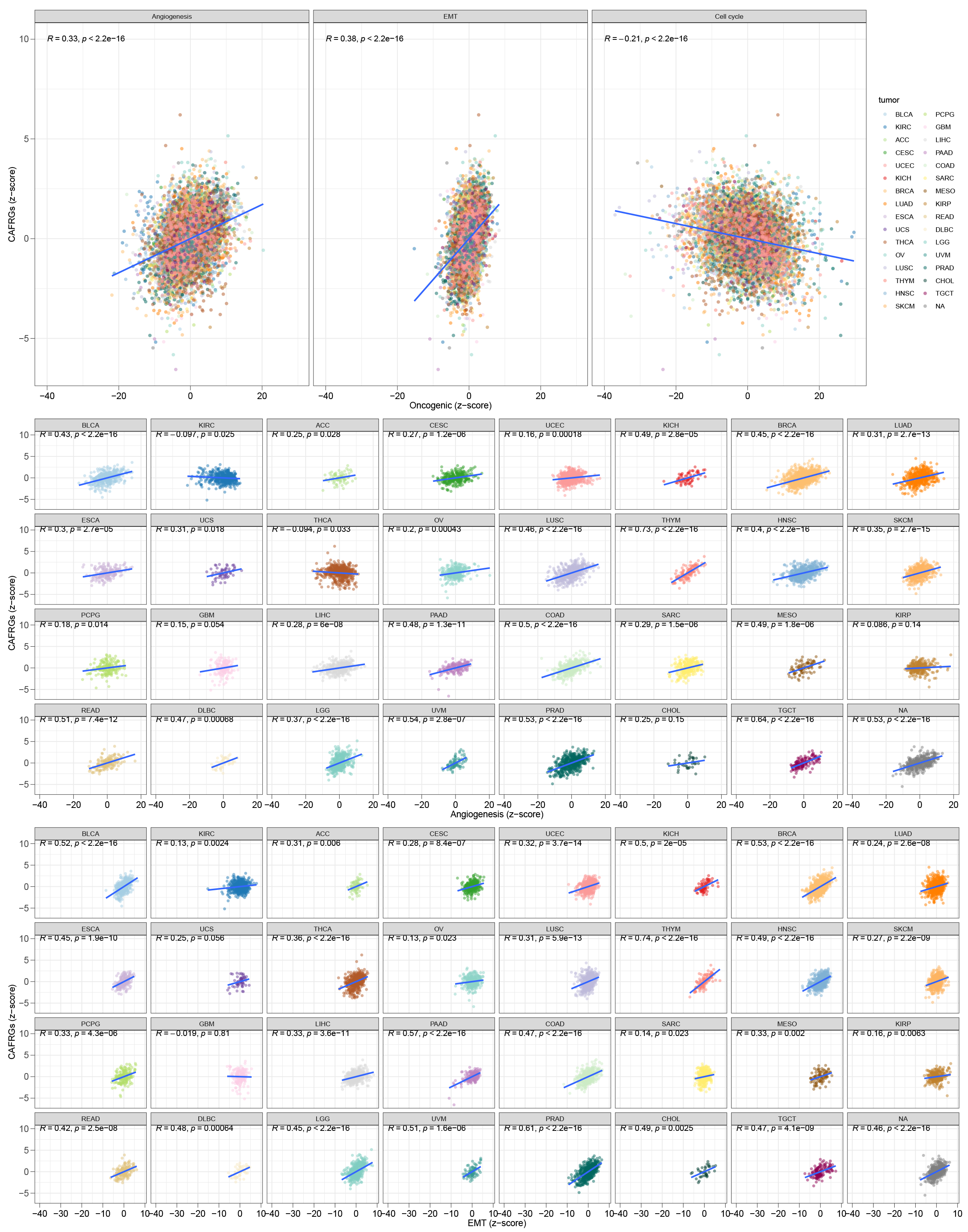

Supplement: Supplementary file 1 — Figures S1–S11 [file JCMM-28-e18262-s001.zip › Figure S11.tif]

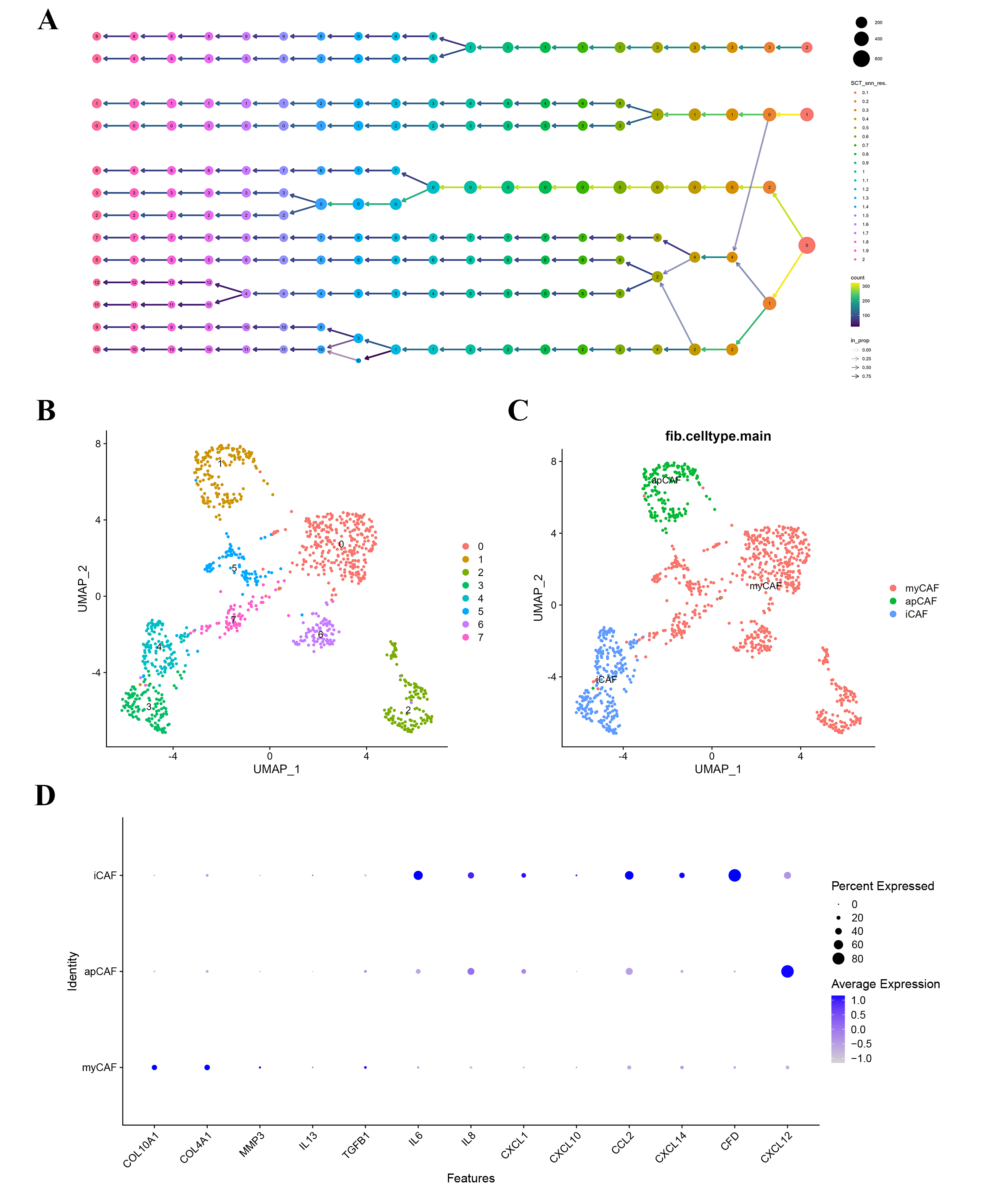

Supplement: Supplementary file 1 — Figures S1–S11 [file JCMM-28-e18262-s001.zip › Figure S2.tif]

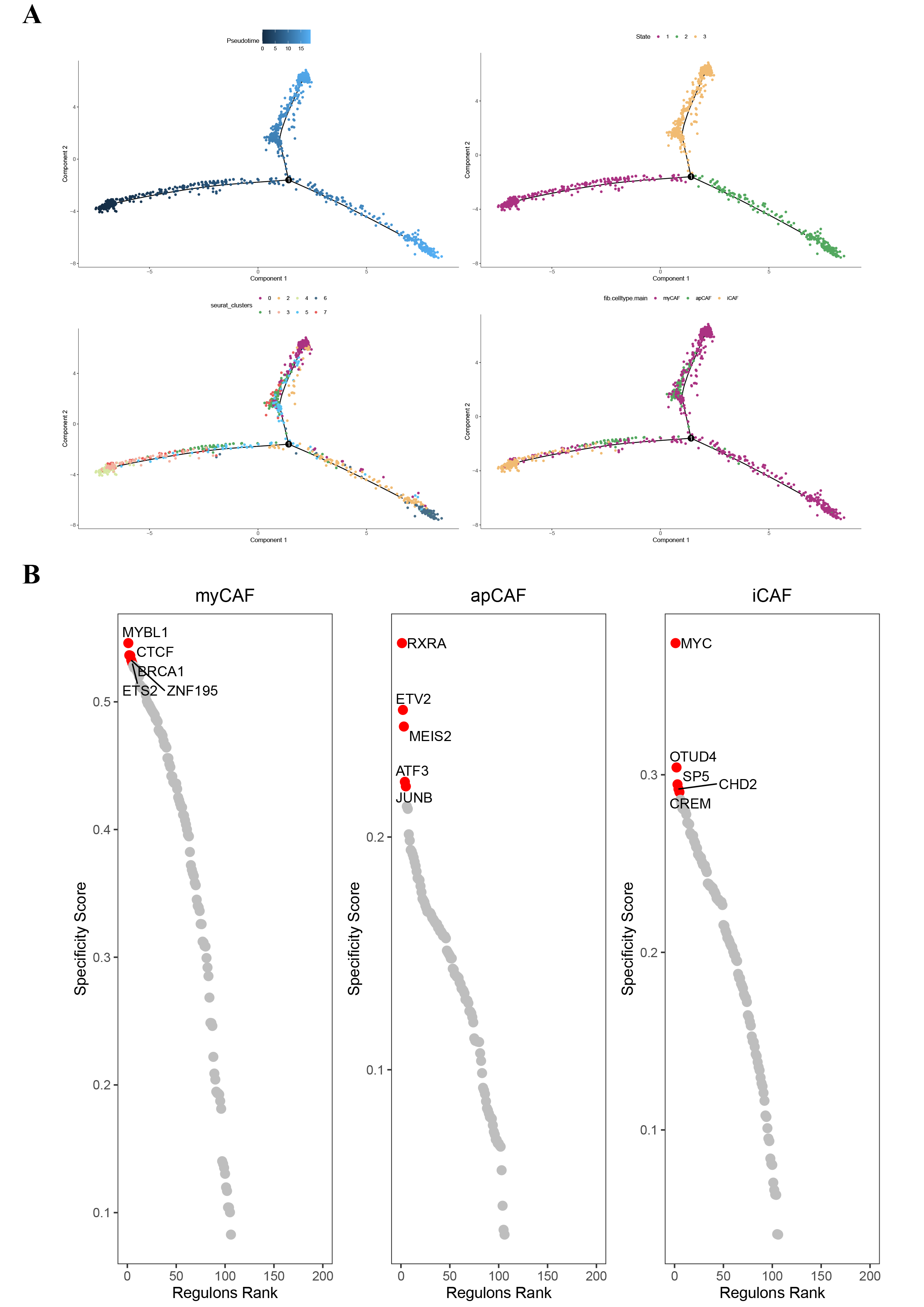

Supplement: Supplementary file 1 — Figures S1–S11 [file JCMM-28-e18262-s001.zip › Figure S3.tif]

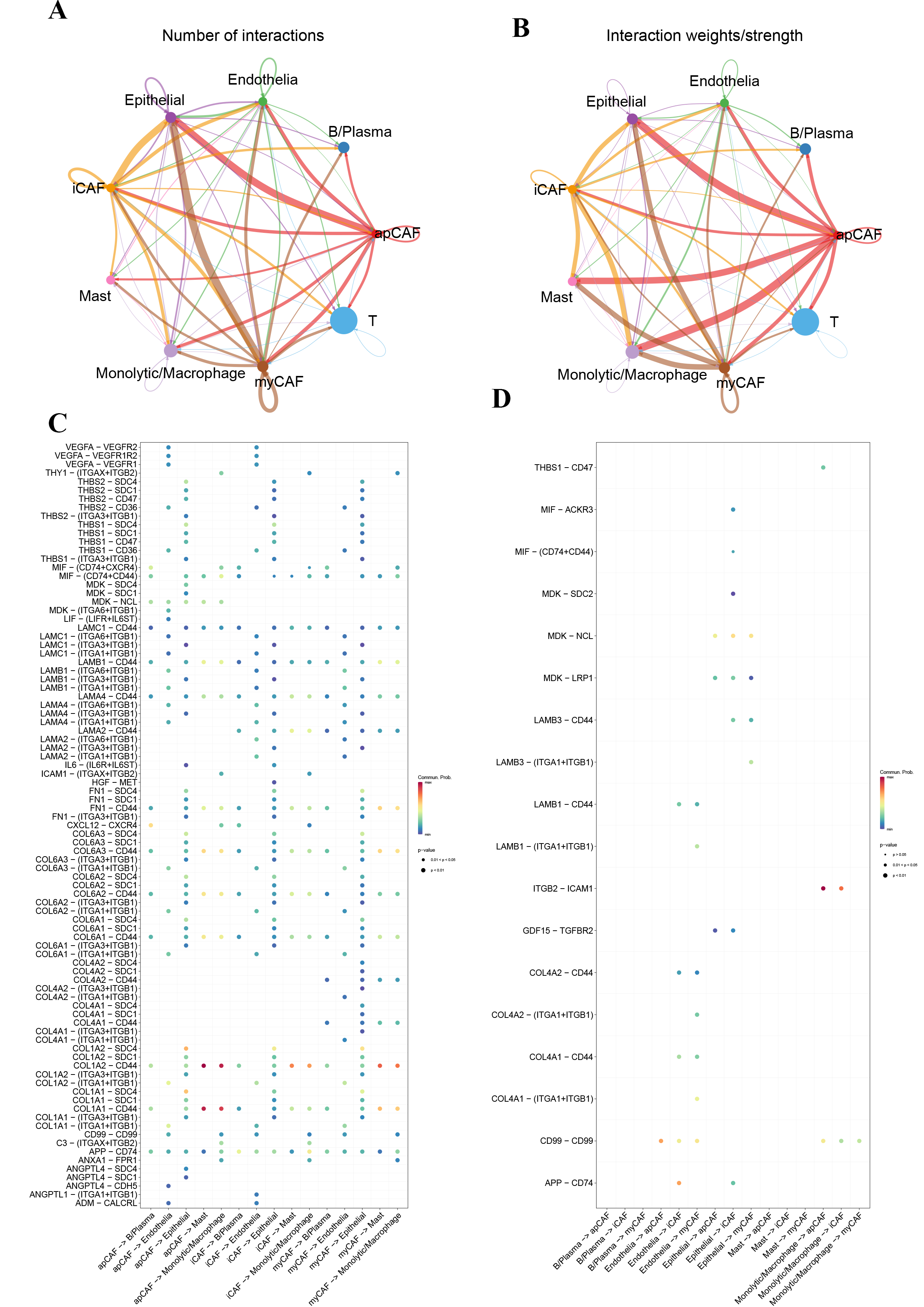

Supplement: Supplementary file 1 — Figures S1–S11 [file JCMM-28-e18262-s001.zip › Figure S4.tif]

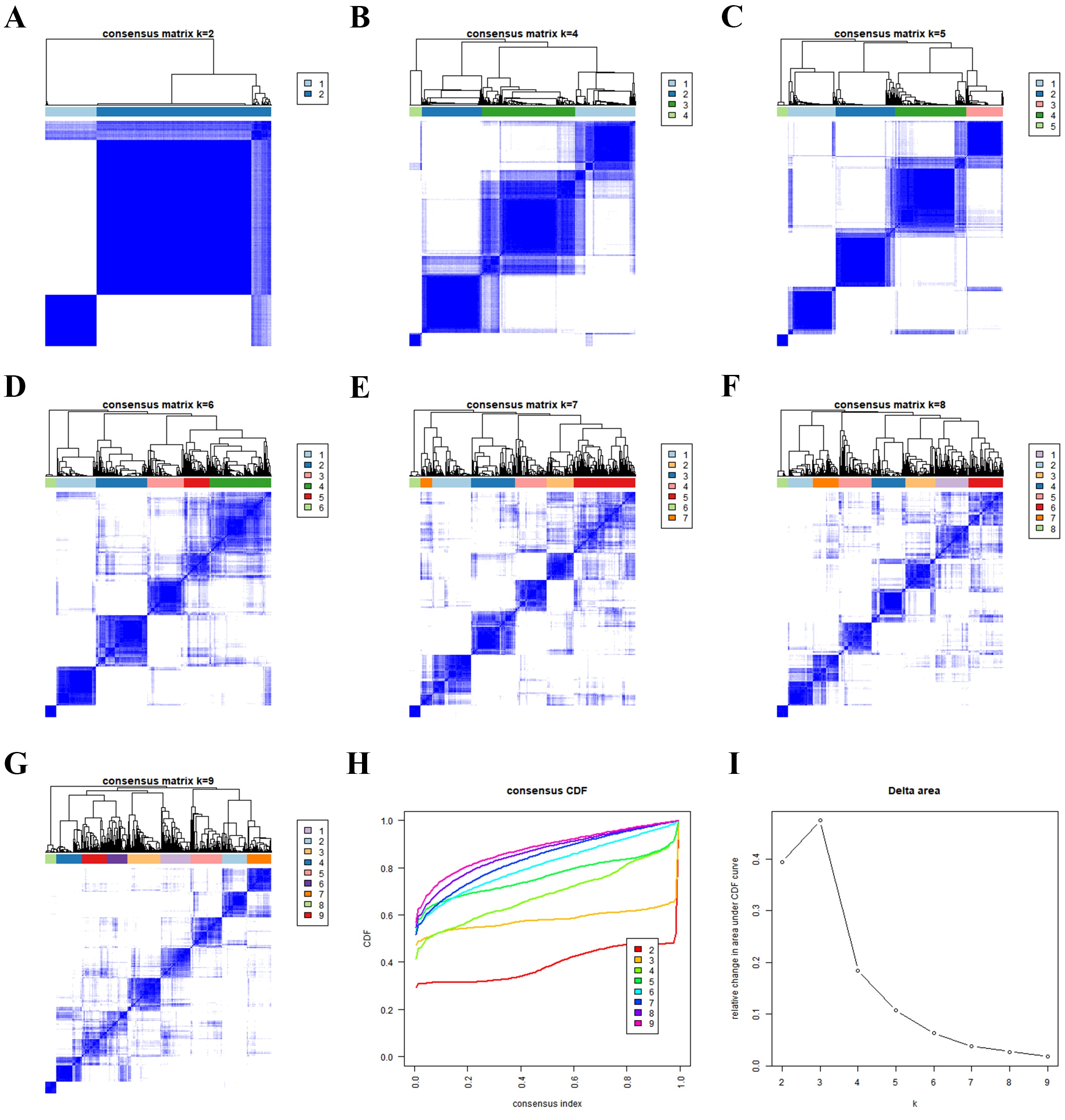

Supplement: Supplementary file 1 — Figures S1–S11 [file JCMM-28-e18262-s001.zip › Figure S5.tif]

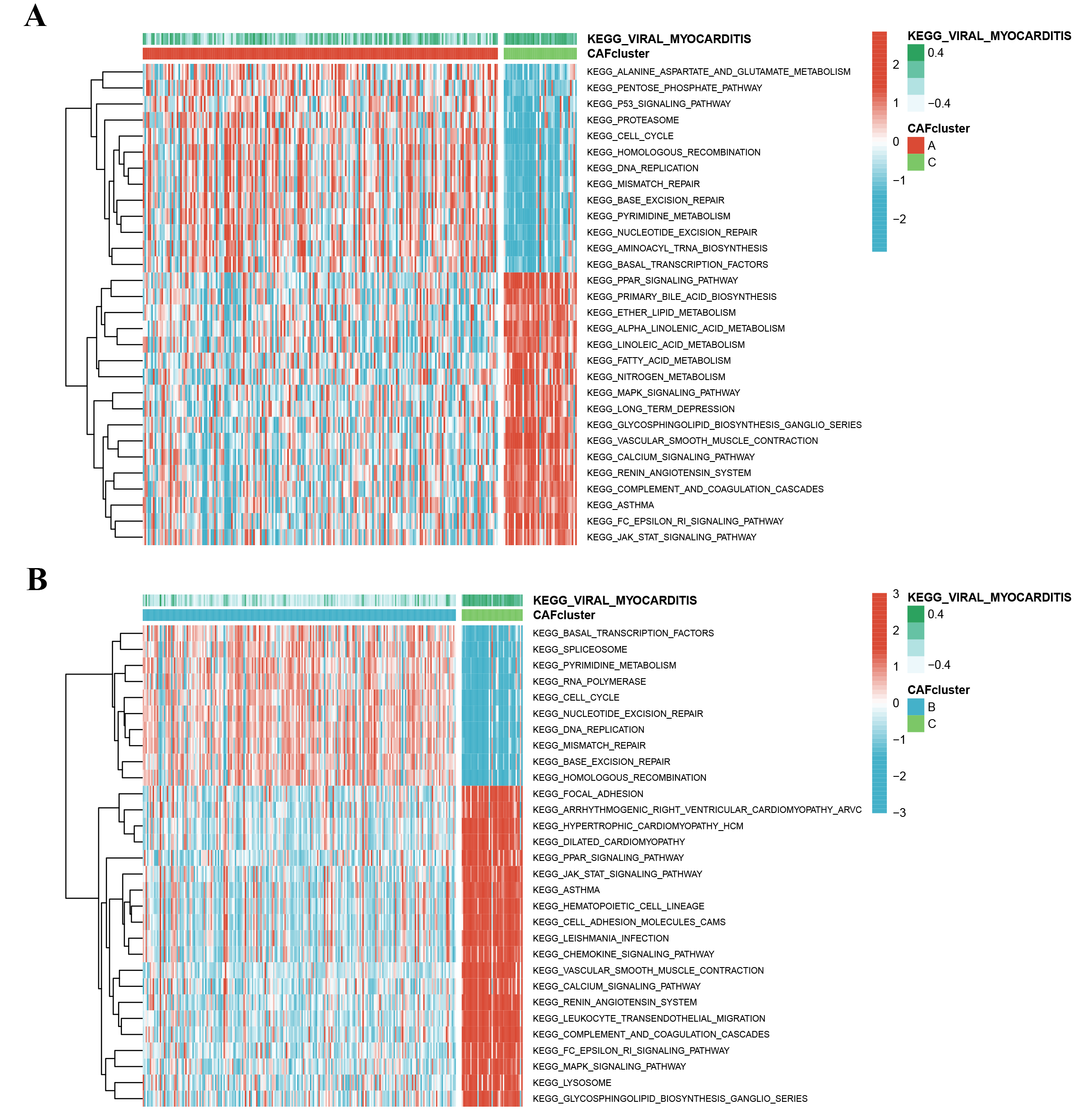

Supplement: Supplementary file 1 — Figures S1–S11 [file JCMM-28-e18262-s001.zip › Figure S6.tif]

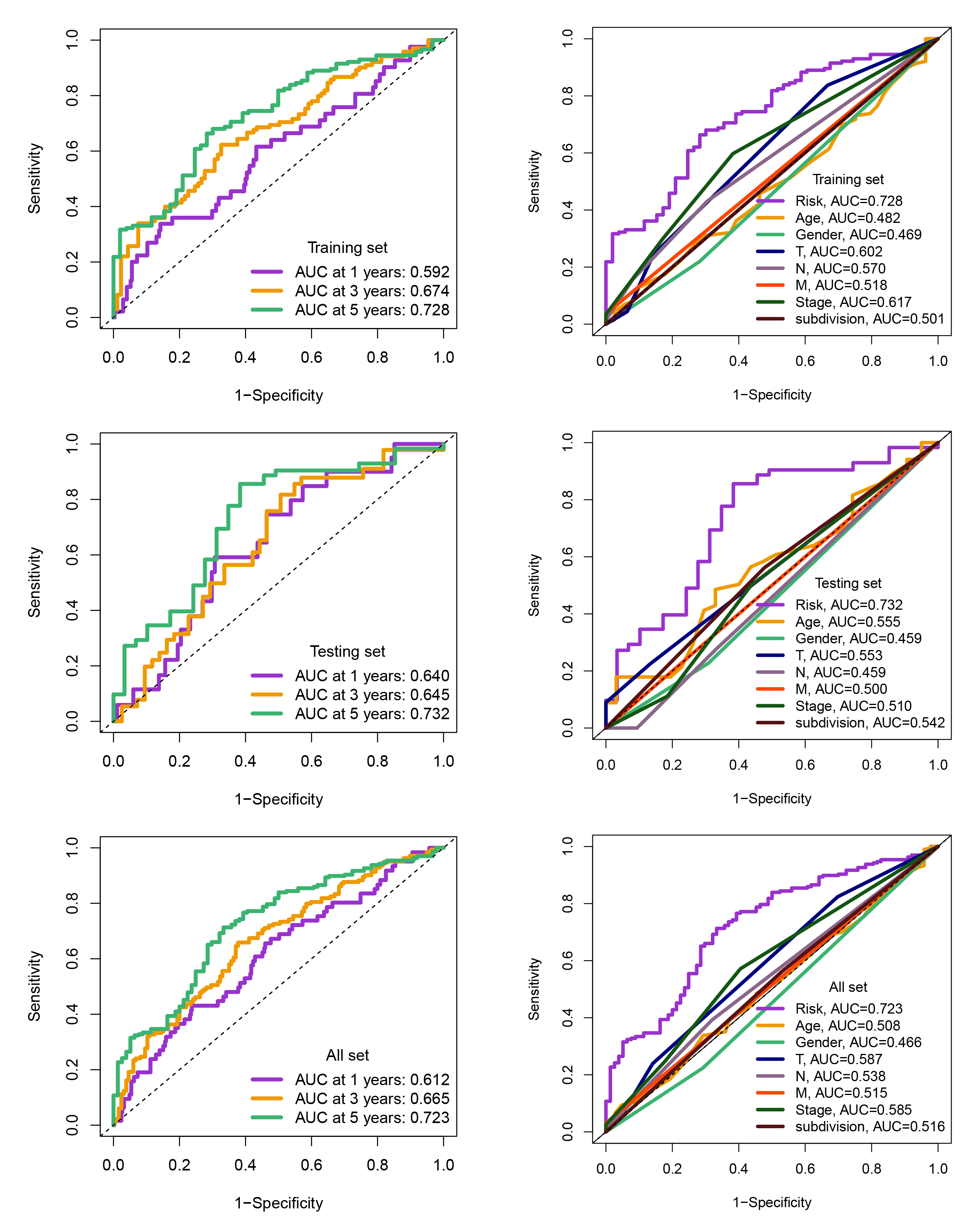

Supplement: Supplementary file 1 — Figures S1–S11 [file JCMM-28-e18262-s001.zip › Figure S7.tif]

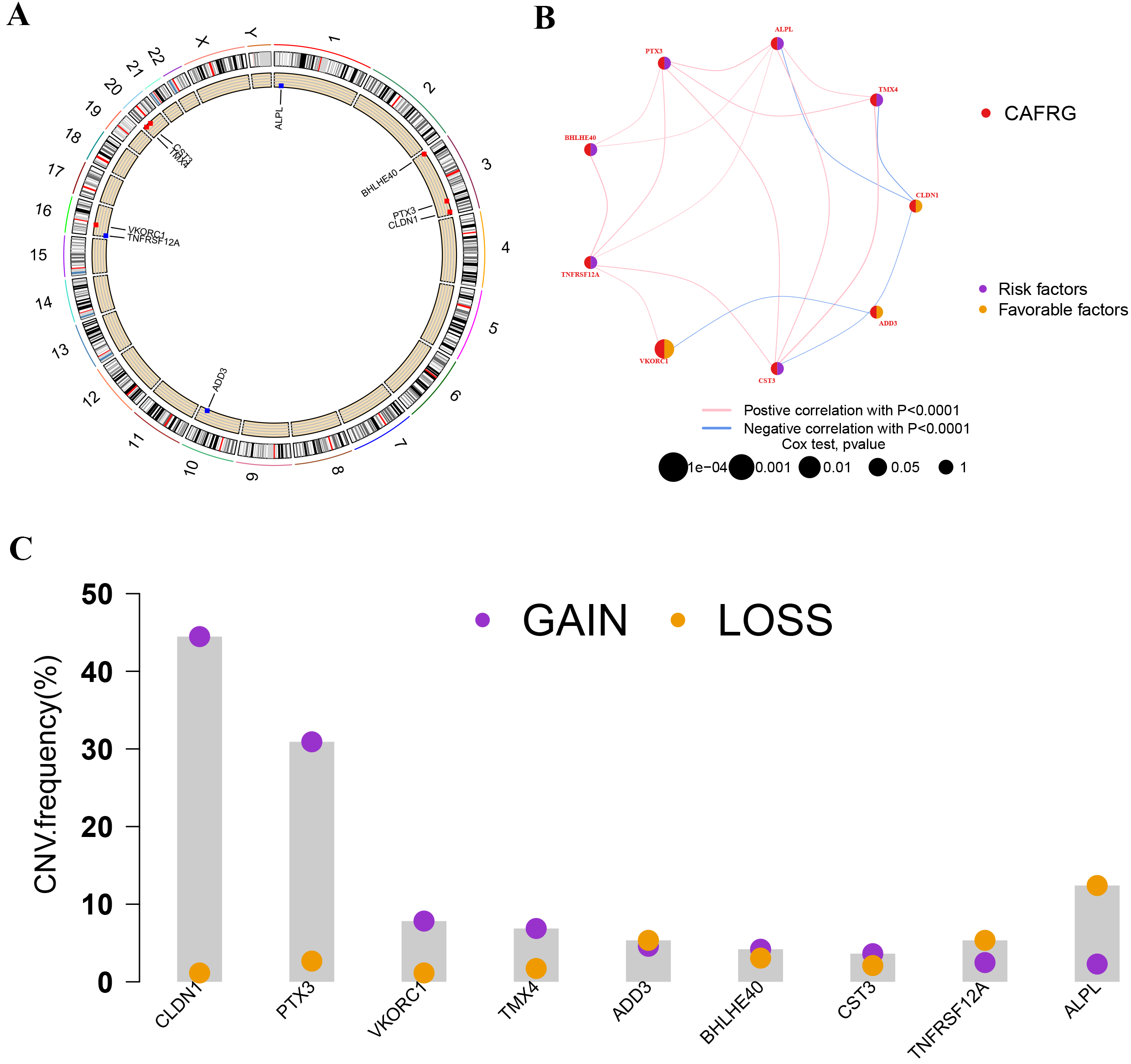

Supplement: Supplementary file 1 — Figures S1–S11 [file JCMM-28-e18262-s001.zip › Figure S9.tif]
